# Chronic neurostimulation of splenic nerve enabled by hydrogel-bioelectronics for wireless electroceutical immunomodulation therapy

**DOI:** 10.1093/nsr/nwaf557

**Published:** 2025-12-05

**Authors:** Wenliang Liu, Qiong Wang, Renyuan Sun, Ming Yang, Ping Wu, Dingke Zhang, Kun Yang, Chong Ma, Chuan Gao, Nanxi Yi, Zhikun Li, Long Wen, Luyao Wu, Xiaokun Li, Jiexiong Feng, Zhouguang Wang, Zhiqiang Luo

**Affiliations:** National Engineering Research Center for Nanomedicine, Research Center for Intelligent Fiber Devices and Equipment, State Key Laboratory of New Textile Materials and Advanced Processing, College of Life Science and Technology, Huazhong University of Science and Technology, Wuhan 430074, China; Department of Pediatric Surgery, Tongji Hospital, Tongji Medical College, Huazhong University of Science and Technology, Wuhan 430030, China; National Engineering Research Center for Nanomedicine, Research Center for Intelligent Fiber Devices and Equipment, State Key Laboratory of New Textile Materials and Advanced Processing, College of Life Science and Technology, Huazhong University of Science and Technology, Wuhan 430074, China; National Engineering Research Center for Nanomedicine, Research Center for Intelligent Fiber Devices and Equipment, State Key Laboratory of New Textile Materials and Advanced Processing, College of Life Science and Technology, Huazhong University of Science and Technology, Wuhan 430074, China; The First Affiliated Hospital of Wenzhou Medical University, Wenzhou 325035, China; National Engineering Research Center for Nanomedicine, Research Center for Intelligent Fiber Devices and Equipment, State Key Laboratory of New Textile Materials and Advanced Processing, College of Life Science and Technology, Huazhong University of Science and Technology, Wuhan 430074, China; National Engineering Research Center for Nanomedicine, Research Center for Intelligent Fiber Devices and Equipment, State Key Laboratory of New Textile Materials and Advanced Processing, College of Life Science and Technology, Huazhong University of Science and Technology, Wuhan 430074, China; National Engineering Research Center for Nanomedicine, Research Center for Intelligent Fiber Devices and Equipment, State Key Laboratory of New Textile Materials and Advanced Processing, College of Life Science and Technology, Huazhong University of Science and Technology, Wuhan 430074, China; National Engineering Research Center for Nanomedicine, Research Center for Intelligent Fiber Devices and Equipment, State Key Laboratory of New Textile Materials and Advanced Processing, College of Life Science and Technology, Huazhong University of Science and Technology, Wuhan 430074, China; National Engineering Research Center for Nanomedicine, Research Center for Intelligent Fiber Devices and Equipment, State Key Laboratory of New Textile Materials and Advanced Processing, College of Life Science and Technology, Huazhong University of Science and Technology, Wuhan 430074, China; National Engineering Research Center for Nanomedicine, Research Center for Intelligent Fiber Devices and Equipment, State Key Laboratory of New Textile Materials and Advanced Processing, College of Life Science and Technology, Huazhong University of Science and Technology, Wuhan 430074, China; Department of Pediatric Surgery, Tongji Hospital, Tongji Medical College, Huazhong University of Science and Technology, Wuhan 430030, China; Department of Pediatric Surgery, Tongji Hospital, Tongji Medical College, Huazhong University of Science and Technology, Wuhan 430030, China; The First Affiliated Hospital of Wenzhou Medical University, Wenzhou 325035, China; Department of Pediatric Surgery, Tongji Hospital, Tongji Medical College, Huazhong University of Science and Technology, Wuhan 430030, China; Hubei Clinical Center of Hirschsprung’s disease and allied disorders, Wuhan 430074, China; The First Affiliated Hospital of Wenzhou Medical University, Wenzhou 325035, China; National Engineering Research Center for Nanomedicine, Research Center for Intelligent Fiber Devices and Equipment, State Key Laboratory of New Textile Materials and Advanced Processing, College of Life Science and Technology, Huazhong University of Science and Technology, Wuhan 430074, China; Department of Pediatric Surgery, Tongji Hospital, Tongji Medical College, Huazhong University of Science and Technology, Wuhan 430030, China

**Keywords:** bioelectronics, electroceuticals, neuromodulation, neural interface, conductive hydrogels

## Abstract

Targeted modulation of the splenic nerve offers a selective alternative to vagus nerve stimulation, avoiding off-target activation of mixed vagal fibers. However, the small diameter and anatomical complexity of the splenic nerve pose great challenges for stable neural interfacing. Here, we report a splenic nerve wireless stimulator (SpNWS) built from stretchable and highly conductive hydrogel for chronic electroceutical immunomodulation therapy. The hydrogel-fiber neural electrodes conform to the geometries of splenic neurovascular bundles (SNVBs) and are secured using a bioadhesive approach, without inducing fibrosis or immune activation in SNVBs. SpNWSs deliver wireless stimulation using subcutaneously implanted hydrogel thin film as battery-free powering electrodes with a capacitive-coupling effect. In a chronic inflammatory bowel disease rat model, splenic nerve stimulation by an SpNWS significantly reduced colitis severity and restored intestinal immune balance by suppressing T_H_1/T_H_17 responses and enhancing T_H_2/T_reg_ activity. This work establishes a bioelectronic platform that combines material compliance, interface adaptability and wireless power delivery to enable organ-specific neuromodulation for electroceutical treatment of refractory diseases.

## Teaser

A wireless, fully implantable hydrogel neurostimulator enables long-term modulation of the splenic nerve to treat inflammatory bowel disease by rebalancing the gut immune system.

## INTRODUCTION

Bioelectronic medicine provides a promising therapeutic approach that modulates the electrical activity of disease-relevant nerves to treat neurological, inflammatory and metabolic disorders, offering an alternative to conventional pharmacological interventions [1–3]. Among its key technologies, vagus nerve stimulation (VNS) has not only demonstrated therapeutic potential for chronic inflammatory diseases such as inflammatory bowel disease (IBD) and rheumatoid arthritis, but has also advanced to clinical applications [4–7]. However, conventional VNS targeting the human cervical vagus trunk, a complex bundle comprising approximately 100 000 heterogeneous fibers, lacks neural specificity [8,9]. This leads to the non-selective activation of both efferent and afferent fibers, often causing side effects such as bradycardia, arrhythmia, vocal cord paralysis and chronic cough [10–12]. To overcome these limitations, recent strategies in bioelectronic medicine have attempted near-organ neuromodulation by stimulating peripheral nerves near specific organs, aiming to enhance selectivity and reduce systemic side effects [13–15].

One representative example of near-organ neuromodulation is splenic nerve stimulation (SNS), which induces norepinephrine (NE) release by splenic nerve axons, thereby suppressing pro-inflammatory cytokine secretion and modulating systemic inflammation [16]. Since the splenic nerve serves as the final common pathway for efferent cholinergic anti-inflammatory signaling, SNS avoids the non-selective engagement of VNS, thereby enabling greater therapeutic efficacy and improved long-term safety [17,18]. Anatomically, the splenic nerve runs alongside the vasculature within the adipose tissue of the splenic ligaments, forming splenic neurovascular bundles (SNVBs). It has a small diameter and predominantly unmyelinated or thinly myelinated fibers, both of which complicate surgical access and increase the risk of neural or vascular injury [19]. Current research on splenic nerve modulation predominantly focuses on acute stimulation paradigms employing destructive surgical nerve dissection with rigid cuff implantation, which is incompatible with chronic clinical requirements and limited in translational potential [16,20–22]. For instance, Mughrabi *et al.* implanted commercial cuff electrodes around the SNVBs of mice, acutely stimulated the splenic nerve, and monitored NE release in real time using fast-scan cyclic voltammetry (CV) [22]. However, the irregular geometries and anatomical variability of SNVBs, including larger proximal and smaller distal ends near the spleen, make it difficult for conventional cuff electrodes designed for uniform anatomy to establish seamless neural interfaces [23]. In addition, commercial cuff electrodes demonstrate critical modulus mismatch with the SNVBs, resulting in potential neural damage from compressive and shear forces during long-term implantation [24]. To enable safe and effective long-term SNS, neural electrodes should possess tissue-matched mechanical properties and optimal conformability.

Conductive hydrogels with excellent mechanical compliance and biocompatibility have emerged as promising neural interface materials for seamless electrical coupling with peripheral nerve trunks [25–27]. In most applications, they are integrated into planar devices that perform well with large, accessible and regularly shaped nerve trunks [28,29]. However, these planar devices have limited ability to conform to the irregular geometries of near-organ nerves, such as SNVBs. In contrast, fiber-shaped materials exhibit 1D morphology and mechanical reconfigurability, enabling active bending, stretching, knotting and weaving into conformal bioelectronic interfaces for neural applications [30–32]. We propose that conductive hydrogel fibers can form intimate neural interfaces on SNVBs without inducing mechanical compression on the splenic nerve and vasculature. However, the flexibility and conductivity of hydrogels are inherently intertwined in a trade-off relationship [33]. Achieving both ultrahigh conductivity and tunable Young’s modulus within a single hydrogel remains a significant challenge.

Besides the advanced neural interface, the fully implantable neurostimulator is another critical component for long-term near-organ neurostimulation. The preclinical evaluation of electroceutical therapy approach is usually performed in rodent models (e.g. rats and mice) [34,35]. The extremely small, submillimeter-scale neuroanatomical structures of these animals impose stringent size constraints on neurostimulators. To meet these constraints, existing research mainly achieves device miniaturization via a battery-free and wireless stimulation design strategy. However, traditional wireless devices, which are typically constructed from rigid modules, restrict the conformability and long-term biocompatibility [36]. Although structures such as wavy, serpentine and 3D helical designs have been developed to improve the mechanical compliance of metallic interconnects, they substantially increase device size and hinder miniaturization [37]. Additionally, the mechanical and electrical mismatches between metal interconnect and hydrogel neural electrodes, including differences in charge transport mechanisms with electrons in metals and ions in hydrogels, lead to elevated interfacial resistance and compromise the long-term stability of metal–hydrogel interfaces under chronic physiological conditions [38,39]. Therefore, the development of soft miniature wireless neurostimulators with all critical components (wireless modules, interconnects and neural electrodes) made of advanced hydrogel materials is highly demanded.

**Figure 1.**
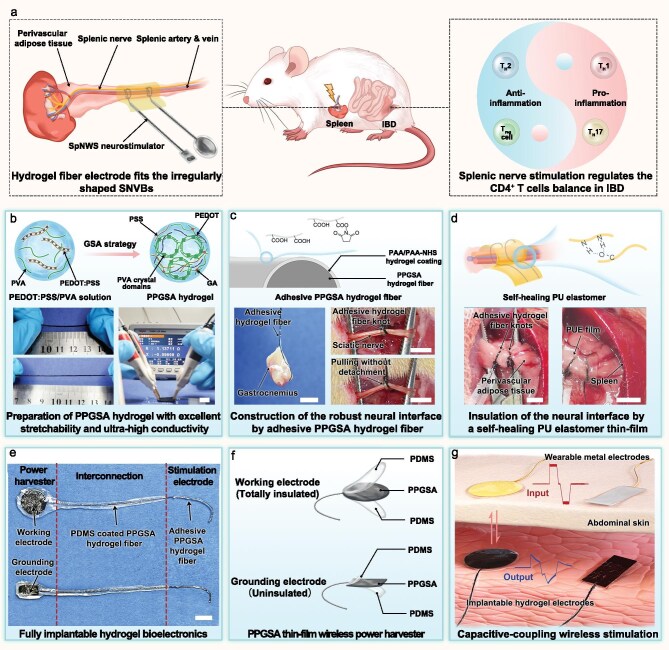
Chronic SNS enabled by hydrogel bioelectronics for wireless electroceutical treatment. (a) Schematic illustration of electroceutical IBD treatment by SpNWS-enabled wireless SNS, which restores intestinal CD4^+^ T cell subsets homeostasis in IBD rats. The fiber-shaped neural electrodes in the SpNWS can be fixed on SNVBs through a wrapping and bioadhesive strategy. (b) Fabrication process of PPGSA hydrogel and experimental demonstrations of its stretchable and highly conductive properties. The PPGSA hydrogel exhibited over 100% breaking elongation, while its thin-film samples showed a resistance of only 1.14 Ω. Scale bars, 1 cm. (c) Structural hierarchy schematics of adhesive PPGSA hydrogel fibers and the demonstration of its tissue-adhesion performance. The carboxyl groups and *N*-hydroxysuccinimide esters conferred adhesive capabilities to PPGSA hydrogel. The adhesive PPGSA hydrogel fibers have robust adhesion to gastrocnemius muscles and sciatic nerves. Scale bars, 5 mm. (d) Surgical procedure diagrams demonstrating the implantation of the SpNWS insulated with self-healing PU elastomer. Scale bar, 5 mm. (e) Photograph of the SpNWS with adhesive PPGSA hydrogel fibers. The SpNWS comprised three functionally integrated components: a neural electrode section made of PPGSA hydrogel fibers; an interconnection segment made of PDMS-encapsulated PPGSA hydrogel fibers; and wireless capacitive-coupling power-harvesting modules, where a circular PPGSA hydrogel film served as a working electrode and a rectangular PPGSA hydrogel film served as a grounding electrode. Scale bar, 5 mm. (f) The hierarchical architecture of the working and grounding electrodes of the SpNWS. The working electrode comprised a PDMS-insulated PPGSA hydrogel film, while the PPGSA hydrogel grounding electrode was partially exposed. (g) Schematic illustration of SpNWS-enabled wireless SNS. The SpNWS’s working electrode and grounding electrode were subcutaneously implanted in the abdomen, with precise alignment and close contact maintained, respectively, with the external power-transmitter electrode and external grounding electrode to support wireless power transmission. The external power-transmitter electrode is a 1-cm-diameter circular Au foil (0.4 μm thick) insulated with polyurethane (PU) films, connected to copper wires for input signal transmission. The grounding electrode is a 5 mm × 10 mm rectangular titanium film (20 μm thick) partially insulated with PU films (exposing approximately 10 mm^2^), connected to copper wires for grounding.

To address these critical challenges in chronic neuromodulation of the splenic nerve, we developed a fully hydrogel-based wireless neurostimulator. For the first time, we demonstrated successful chronic electroceutical therapy of IBD in a rat model with a splenic nerve wireless stimulator (SpNWS) (Fig. 1a). A glutaraldehyde (GA) crosslinking–salt treatment–wet annealing (GSA) strategy was explored to prepare the poly(3,4-ethylenedioxythiophene):poly(styrenesulfonate) (PEDOT:PSS)-incorporated polyvinyl alcohol (PVA) conductive hydrogel (PPGSA), which exhibits ultrahigh conductivity and excellent stretchability (Fig. 1b). The unique fiber-shaped PPGSA hydrogel served as the neural electrodes, achieving seamless contact with SNVBs via a bioadhesive coating (Fig. 1a–d). After bioadhesive fixation, PPGSA fibers were insulated with a self-healing polyurethane elastomer (PUE) film (Fig. 1d). An SpNWS is a fully implantable, battery-free device comprising neural stimulation electrodes made of bioadhesive PPGSA fibers, stretchable interconnects made of PPGSA fibers, and capacitive-coupled wireless-powering modules made of PPGSA films (Fig. 1e). The working and grounding electrodes of SpNWSs are a fully insulated circular and a partially exposed rectangular PPGSA film, respectively (Fig. 1f). In the rat IBD model, subcutaneously implanted SpNWS modules were capacitively coupled with external wearable power-delivery electrodes, enabling wireless power transfer for neurostimulation (Fig. 1g). Overall, this hydrogel bioelectronics establishes an advanced framework for constructing complex organ-proximal neural interfaces, laying a foundation for precision neuromodulation in bioelectronic medicine.

## RESULTS AND DISCUSSION

### Design and characterization of PPGSA hydrogel

Although PEDOT:PSS is a widely used conductive polymer in bioelectronics, its electrical performance is often compromised by the excessive presence of insulating PSS, which acts as a dispersant for PEDOT but hinders charge transport. Post-treatment strategies such as dimethyl sulfoxide (DMSO) soaking, ionic liquid (IL) exposure, and thermal annealing have been shown to enhance conductivity of PEDOT:PSS by promoting PEDOT aggregation and phase separation [40]. Meanwhile, these strategies can construct an interconnected network of PEDOT:PSS nanofibers, thereby yielding pure PEDOT:PSS hydrogels [41,42]. However, these PEDOT:PSS hydrogels exhibit poor mechanical properties, particularly a high modulus that is incompatible with soft neural tissue interfaces. To overcome this limitation, PEDOT:PSS is often embedded in hydrogel matrices such as PVA to improve stretchability and reduce modulus [43]. However, the resulting PEDOT:PSS/PVA composite hydrogels introduce a new set of trade-offs. Enhancing PVA crystallinity, commonly used to improve mechanical strength, can disrupt the percolating pathways of PEDOT domains, thereby reducing electrical conductivity.

**Figure 2.**
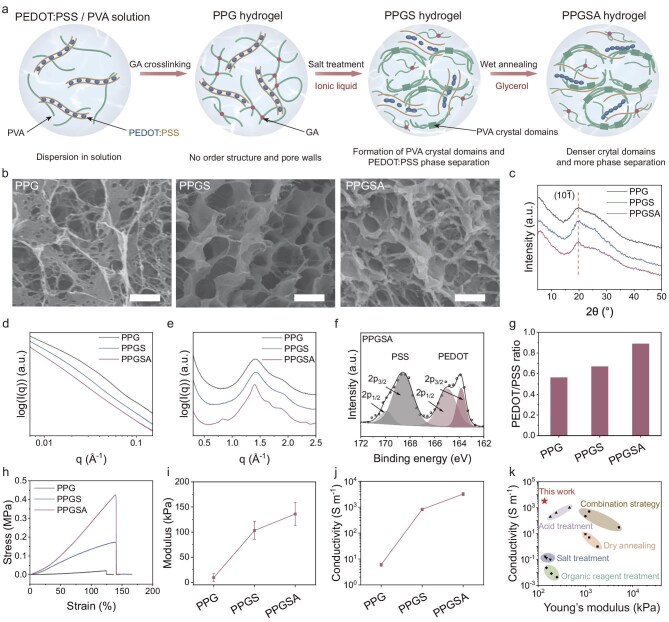
Synthesis and characterization of PPGSA hydrogel. (a) Schematic illustration of the structural changes of PVA and PEDOT:PSS during PPGSA hydrogel fabrication. PPG hydrogel exhibited weak PVA cross-linking and homogeneous PEDOT:PSS dispersion. PPGS hydrogel featured emergent PVA crystalline domains and initial PEDOT:PSS phase separation. PPGSA hydrogel showed improved PVA crystallization and enhanced PEDOT:PSS separation. (b) Representative SEM images of PPG, PPGS and PPGSA hydrogels. Scale bars, 1 μm. (c) XRD spectra of PPG, PPGS and PPGSA hydrogels. The characteristic peak at 2θ = 19.8° corresponds to diffraction from PVA’s ($10\bar{1}$) crystallographic plane. (d) SAXS spectra of PPG, PPGS and PPGSA hydrogels. PPGS and PPGSA hydrogel curves were nearly parallel, while PPG hydrogel exhibited smaller slopes at small q-values. (e) WAXS spectra of PPG, PPGS and PPGSA hydrogels. PPGSA hydrogel exhibited additional peaks at 0.8 and 1.6 Å^−1^. (f) XPS characterization of PPGSA hydrogel. PPGSA hydrogel exhibited S_2p_ characteristic peaks of sulfur atoms in PEDOT (161–167 eV) and PSS (167–171 eV). (g) Quantitative analysis of normalized PEDOT/PSS ratios in PPG, PPGS and PPGSA hydrogels. The PEDOT/PSS ratios were calculated based on the integrated area ratio of sulfur peaks from PEDOT and PSS in XPS peak deconvolution. (h) Stress–strain curves of PPG, PPGS and PPGSA hydrogels. All the three hydrogels showed comparable fracture strain (∼150%), while PPGSA hydrogel exhibited superior fracture stress (∼0.4 MPa). (i) Young’s modulus of PPG, PPGS and PPGSA hydrogels. The Young’s modulus of PPGSA hydrogel is about 130 kPa, keeping at tissue-matched modulus range (*n* = 3 independent hydrogel samples). (j) Electrical conductivity of PPG, PPGS and PPGSA hydrogels. The electrical conductivity of PPGSA hydrogel is about 3 × 10^3^ S m^−1^ (*n* = 3 independent hydrogel samples). (k) Comparative analysis of PPGSA hydrogel and state-of-the-art PEDOT:PSS/PVA hydrogels in terms of conductivity and Young’s modulus. Compared to existing PEDOT:PSS/PVA hydrogels, PPGSA hydrogel exhibits enhanced electrical conductivity and tissue-compliant softness. Data are presented as mean ± standard deviation in (i) and (j).

To resolve the trade-off between electrical conductivity and mechanical compliance, we developed a GSA strategy to fabricate PPGSA hydrogel with finely tuned microstructure. The fabrication of PPGSA hydrogel proceeded through three critical phases (Fig. 2a). First, PEDOT:PSS and PVA were mixed, crosslinked with GA, and subjected to freeze–thaw cycles to form a loosely crosslinked hydrogel (PPG), establishing a soft, hydrated network for subsequent modulation. Second, PPG was treated with 1-butyl-3-methylimidazolium tetrafluoroborate ([BMIM]BF_4_, an IL)/DMSO solution (1:2 v/v). The ionic liquid facilitated PVA crystallization by promoting hydrogen bonding, while DMSO partially dissolved nascent crystalline regions, slowing down the crystallization process. This controlled dynamic allowed sufficient time for PEDOT:PSS phase separation and domain reorganization. The resulting hydrogel (PPGS) exhibited a porous structure with enhanced PEDOT connectivity and emerging crystalline PVA domains. Finally, PPGS underwent glycerol-mediated wet annealing at 130°C, where glycerol functioned as both a plasticizer and a moisture-retaining agent. This step further enhanced PVA crystallinity and stabilized PEDOT-rich domains without compromising flexibility. The resulting PPGSA hydrogel exhibited well-organized phase separation, improved mechanical robustness, and ultrahigh conductivity with tissue-compliant softness.

To characterize the structural evolution during the PPGSA hydrogel fabrication process, we conducted multiscale analyses including morphology, crystallinity, nanoscale ordering and chemical composition. Scanning electron microscopy (SEM) analysis revealed distinct microstructural evolution: (i) the PPG hydrogel lacked defined pore walls, consistent with its low PVA crosslinking density; (ii) the PPGS hydrogel exhibited clearly visible pores (∼1 μm diameter) with well-developed pore walls, attributable to increased PVA crosslinking induced by salt treatment; and (iii) the PPGSA hydrogel retained a similar pore size to PPGS but showed thickened pore walls, resulting from wet annealing-induced enhancement of PVA crystallinity (Fig. 2b) [44]. These morphological changes were corroborated by the X-ray diffraction (XRD) findings. The diffraction peak corresponding to the $(10\bar{1}$) crystallographic plane of PVA (2θ = 19.8°) appeared broad in the PPG hydrogel, became sharper in the PPGS hydrogel, and reached maximal sharpness in the PPGSA hydrogel, indicating a progressive increase in PVA crystallinity throughout the fabrication process (Fig. 2c) [45].

Small-angle X-ray scattering (SAXS) provided additional validation of interfacial evolution processes, as evidenced by the steeper slope in the low q region (0.007 < q < 0.03 Å^−1^) for PPGS and PPGSA compared to PPG. This observation suggests a reduction in structural defect density and a smoother pore wall following [BMIM]BF_4_/DMSO treatment (Fig. 2d) [46]. Wide-angle X-ray scattering (WAXS) revealed that wet annealing induced hierarchical molecular ordering in PPGSA hydrogel. Compared to PPG and PPGS, the PPGSA hydrogel displayed an emergent diffraction peak at 0.8 Å^−1^, attributed to lamellar stacking of PVA chains, and a strengthened signal at 1.6 Å^−1^, corresponding to π–π stacking of PEDOT (Fig. 2e) [47]. These features link structural compaction to increased crystallinity.

To characterize the phase separation behavior of PEDOT:PSS within the hydrogel matrix, X-ray photoelectron spectroscopy (XPS) was used to quantify the PEDOT/PSS ratios in PPG, PPGS and PPGSA hydrogels. All three hydrogels exhibited characteristic S_2p_ peaks corresponding to sulfur atoms in PEDOT (161–167 eV) and PSS (167–171 eV) (Fig. 2f and Fig. S1) [48]. Peak deconvolution and spectral integration revealed a gradual increase in the PEDOT/PSS ratios across the three hydrogels, rising from 0.56 in PPG to 0.89 in PPGSA (Fig. 2g). Raman spectroscopy further supported the XPS results by showing a gradual narrowing of C=C stretching peak across PPG, PPGS and PPGSA, indicative of progressively enhanced π–π stacking of PEDOT chains (Fig. S2) [49]. Together, these findings suggest that [BMIM]BF_4_/DMSO treatment initiates PEDOT:PSS phase separation, while subsequent wet annealing reinforces this phase-segregated microstructure.

### Mechanical and electrical properties of PPGSA hydrogel

To investigate the property changes during PPGSA hydrogel fabrication, we performed tensile and conductivity tests on PPG, PPGS and PPGSA hydrogels. All three hydrogels exhibited comparable breaking elongation (∼140%), but the PPGSA hydrogel showed higher tensile toughness (287.52 ± 47.86 kJ m^−3^) than PPGS (154.12 ± 73.34 kJ m^−3^) and PPG (13.43 ± 2.31 kJ m^−3^) (Fig. 2h and Fig. S3). The PPGSA hydrogel also exhibited a significantly higher modulus (136.00 ± 23.06 kPa) than the moduli of PPGS (103.17 ± 17.84 kPa) and PPG hydrogels (9.36 ± 7.78 kPa) (Fig. 2i). The PPGSA hydrogel showed improved conductivity (3236.62 ± 423.37 S m^−1^), surpassing PPGS (821.98 ± 94.45 S m^−1^) and PPG (6.06 ± 0.84 S m^−1^) (Fig. 2j and Fig. S4). Moreover, the PPGSA hydrogel fiber exhibited minimal change in resistance upon stretching, with less than 10% increase in resistance at 20% strain, making it well suited for the deformation requirements of implantable devices (Fig. S5). The PPGSA hydrogel prepared via the GSA strategy exhibited robust mechanical properties suitable for *in vivo* implantation and exceptional conductivity, together surpassing the overall performance of previously reported PEDOT:PSS/PVA hydrogels (Fig. 2k) [50–56].

To investigate the regulatory effects of the critical [BMIM]BF_4_/DMSO treatment and wet annealing steps of our GSA strategy on the properties of PEDOT:PSS/PVA hydrogels, we evaluated the tensile properties and conductivity of PPGS hydrogels fabricated with varying IL/DMSO ratios and PPGSA hydrogels prepared under different annealing durations. For PPGS hydrogels, the Young’s modulus gradually increased with rising IL concentrations, reaching a maximum of 266.00 ± 21.66 kPa under pure IL treatment (Fig. S6). For PPGSA hydrogels, the Young’s modulus increased progressively with annealing time, attaining 188.67 ± 58.18 kPa, in the 30-min group (Fig. S7). Conductivity tests showed PPGS hydrogels reached a plateau (∼900 S m^−1^) at IL:DMSO = 1:2, whereas PPGSA hydrogels achieved a plateau (∼3000 S m^−1^) after 10-min annealing (Fig. S8). Based on considerations of enhancing conductivity while maintaining low modulus, the optimal GSA parameters were determined as IL:DMSO = 1:2 with 10-min wet annealing.

To demonstrate the advantages of wet annealing over conventional dry annealing, we evaluated the tensile properties and conductivity of PPGSA hydrogels prepared using water, DMSO and glycerol as annealing solvents. Both water and DMSO rapidly evaporated at typical annealing temperatures (>100°C), inducing internal stress and resulting in wrinkled hydrogels, whereas glycerol ensured uniform annealing and produced a flat-surfaced hydrogel (Fig. S9a). Furthermore, PPGSA hydrogels annealed with water or DMSO exhibited reduced fracture strain, a two-orders-of-magnitude increase in Young’s modulus and no significant conductivity improvement (Fig. S9b–d). These results validate the superiority of wet annealing in fabricating PEDOT:PSS/PVA hydrogels with high conductivity and tunable modulus.

We further characterized the compressive, shea, and swelling properties of PPGSA hydrogel. The compressive modulus of PPGSA hydrogel reached 219.33 ± 12.50 kPa, surpassing the compressive moduli of both PPGS and PPG hydrogels (Fig. S10). This high compressive modulus is likely attributable to pore compression-induced densification and the enhanced contribution of PVA crystalline domains during loading. PPGSA hydrogel exhibited a maximum strain in the linear viscoelastic region (LVR) of ∼0.05% and a complex shear modulus of 11.60 ± 0.42 kPa, markedly lower than the complex shear moduli of PPGS and PPG hydrogels (Figs S11 and S12). This reduced shear modulus is likely attributable to the shear-alignment of PVA lamellar crystals and interfacial slippage during shearing. Notably, all mechanical moduli of PPGSA hydrogel resided within the kPa range, achieving tissue-level mechanical compatibility. Furthermore, the PPGSA hydrogel exhibited key attributes for implantation: high water content (>80%), low post-equilibrium swelling (<10% diameter change in phosphate-buffered saline (PBS) at 37°C), and exceptional long-term stability with only negligible mass loss (<2% over 5 weeks) (Fig. S13). These properties of PPGSA hydrogel collectively meet the critical requirements for stable bioelectronic interfaces, underscoring its suitability for chronic application.

### Construction and device performance of SpNWSs

The research field of wireless neuromodulation offers various options for bioelectronic therapy, including optical, thermal, mechanical, chemical and magnetic methods [3,57,58]. However, when targeting the splenic nerve, several approaches present significant limitations. Optical and thermal techniques typically require genetic modification or external hardware, while mechanical stimulation may cause tissue damage. Chemical approaches face challenges in precise dosing control, and magnetic systems often depend on multi-turn coils that limit miniaturization. In comparison, capacitive coupling provides a particularly suitable solution. It enables a miniaturized, conformal interface that maintains stable performance under organ movement without introducing thermal or mechanical risks, thereby meeting the specific requirements for chronic splenic nerve stimulation. In our previously reported wireless neurostimulation system, capacitive coupling was implemented using rigid molybdenum electrodes, which lacked mechanical compliance with soft tissue [59]. Here, we present a fully soft SpNWS that replaces metal components with conductive PPGSA hydrogels while retaining wireless capacitive-coupling capability. The SpNWS comprises two components: a working electrode module and a grounding electrode module. For the working electrode module, a template-fabricated PPGSA hydrogel fiber (approximately 300 μm in diameter) serves as the neural electrode, while a disk-shaped PPGSA hydrogel film (1 cm in diameter, approximately 200 μm in thickness) with polydimethylsiloxane (PDMS) coating functions as the working electrode, both interconnected by a PDMS-insulated PPGSA hydrogel fiber (Fig. S14a and b). Similarly, for the grounding electrode module, a PPGSA hydrogel fiber acts as the neural electrode, and a rectangular PPGSA hydrogel film (3 mm × 8 mm, approximately 200 μm in thickness) partially insulated with PDMS (exposing approximately 6 mm^2^) serves as the grounding electrode, both interconnected by a PDMS-insulated PPGSA hydrogel fiber (Fig. S14a and b).

**Figure 3.**
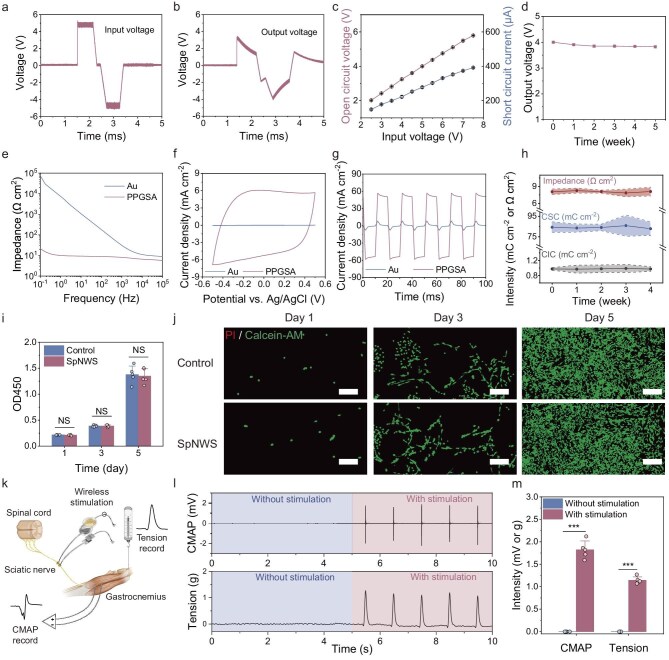
Device performances and biosafety of the SpNWS. (a) Input voltage pulse waveform of the external power-transmitter electrode. The input voltage adopted a 1 MHz sinusoidal carrier-modulated pulsed rectangular wave (5 V, 2 ms pulse width, 1 s pulse interval). (b) Output voltage pulse waveform generated by the working electrode of the SpNWS. (c) Open-circuit voltages and short-circuit currents generated by the working electrode under varying input voltages (*n* = 5 independent working electrodes). (d) Output voltage stability of the working electrode under 37°C PBS-immersed conditions for 4 weeks (*n* = 5 independent working electrodes). (e–g) Electrochemical impedance spectroscopy (e), CV (f) and CIC characteristic (g) of PPGSA hydrogel fiber electrodes versus Au electrodes. Compared with Au electrodes, PPGSA electrodes showed lower impedance, higher CSC and enhanced CIC. (h) Long-term electrochemical stability of PPGSA electrodes in PBS at 37°C over 4 weeks (*n* = 5 independent PPGSA electrodes). (i) Viability of PC12 cells co-cultured with the SpNWS extract at 1, 3 and 5 days. PC12 cells exhibited similar proliferation kinetics between the SpNWS and control groups (*n* = 5 independent SpNWS samples). (j) Representative live–dead staining of PC12 cells co-cultured with the SpNWS extract at 1, 3 and 5 days. PC12 cells exhibited comparable cytomorphological characteristic and population density between the SpNWS and control groups. Scale bars, 100 μm. (k) Schematic diagram recording gastrocnemius CMAP and tension when the SpNWS stimulated the sciatic nerve in rats via capacitive-coupling wireless power transfer. (l) CMAP and tension response variations in gastrocnemius muscle to SpNWS-enabled sciatic stimulation. (m) Quantitative comparison of CMAP and tension response in gastrocnemius muscle with versus without sciatic nerve stimulation. SpNWS achieved wireless modulation of the sciatic nerve through a capacitive coupling effect (*n* = 5 independent animals). Data are presented as mean ± standard deviation in (c), (d), (h), (i) and (m) and were analyzed by one-way ANOVA first, and then by the Tukey’s *post hoc* test. NS, not significant, ***P ≤ 0.001. (i) *P*_day1_ = 0.0962, *P*_day3_ = 0.9415, *P*_day5_ = 0.7775. (m) *P*_CMAP_ = 1.3441 × 10^−7^, *P*_Tension_ = 9.9349 × 10^−8^.

By inputting a precisely modulated biphasic pulse waveform (±5 V amplitude, 2 ms pulse width, single-cycle burst, 1 s pulse interval) with a sinusoidal carrier waveform (±0.5 V, 1 MHz) in the external Au-foil power-transmitter electrode, the SpNWS achieved wireless biphasic pulse transfer with only ∼20% reduction in the voltage peak (Fig. 3a and b). Increasing the input voltage applied to the external transmitter electrode enhanced both the open-circuit voltage and short-circuit current induced by the working electrode of the SpNWS, thus showing controllable output characteristic (Fig. 3c). Under a ±5 V input voltage, the output current of the SpNWS decreased with increasing load, while its output voltage increased with rising load (Fig. S15a). The peak output power of the SpNWS occurred at loads of 2–10 kΩ (corresponding to the typical resistance range of the splenic neurovascular bundles), confirming its suitability for SNS (Fig. S15b). The SpNWS maintained >90% output voltage (±5 V input) during 5-week immersion in *in vitro*-simulated physiological environments, showing stable wireless stimulation performance (Fig. 3d). Notably, with an external input voltage as low as 5 V, the SpNWS implanted in SNVBs generated an internal voltage of up to 1.6 V and a current of 220 μA, sufficient to effectively activate the SNVBs (Fig. S15c and d). Moreover, increasing the coupling distance to 11 mm led to negligible output reduction (±5 V input), while a coupling efficiency of >60% was well preserved with a misalignment of up to 2 mm (Fig. S16a and b).

To evaluate the neuromodulation capability of the PPGSA hydrogel fiber electrode, we systematically investigated its electrochemical properties, including electrochemical impedance, charge storage capacity (CSC), and charge injection capacity (CIC). A conventional Au electrode was used as the control for comparison. Across 0.1 Hz to 1 MHz, the PPGSA electrode showed lower impedance than the Au electrode, with values of 8.06 ± 0.49 Ω cm^2^ versus 30.98 ± 0.40 Ω cm^2^ at 1 kHz (Fig. 3e and Fig. S17a). Phase angle analysis revealed that the PPGSA electrode approached 0° within the mid-frequency range (1 Hz–1 kHz), in contrast to (−70°) for the Au electrode, indicating a predominant resistive impedance characteristic (Fig. S17b). CV analysis showed that the CSC of the PPGSA electrode (84.293 ± 5.294 mC cm^−2^) was over two orders of magnitude higher than that of the Au electrode (0.103 ± 0.002 mC cm^−2^) (Fig. 3f and Fig. S17c). Under biphasic pulsed stimulation (±0.5 V, 20 ms), the PPGSA electrode achieved a CIC of 972.60 ± 34.93 μC cm^−2^, exceeding the Au electrode (50.75 ± 3.66 μC cm^−2^) by approximately 19-fold (Fig. 3g and Fig. S17d). Both the CSC and CIC remained stable in the PPGSA electrode after 5000 consecutive CV cycles and charge injection cycles, indicating robust operational stability (Fig. S18). Long-term soaking in PBS at 37°C for 4 weeks resulted in no detectable changes in impedance, CSC or CIC, confirming the sustained electrochemical stability of the PPGSA electrode (Fig. 3h).

### Biocompatibility and stimulation efficacy of the SpNWS

The biocompatibility of PPGSA hydrogel is primarily determined by residual small molecules from the fabrication process, particularly GA, DMSO and IL. To address this crucial aspect, we implemented a systematic purification protocol where each preparation step was followed by repeated washing with excess deionized water. Ultraviolet–visible (UV–vis) spectroscopic analysis confirmed the effectiveness of this rigorous washing procedure in eliminating residual GA, DMSO and IL, thereby ensuring the biosafety of the final hydrogel material (Fig. S19). The *in vitro* biocompatibility of the SpNWS was assessed using CCK-8 assays and live/dead cell staining in PC12 neuronal cells and L929 fibroblasts co-cultured with SpNWS extracts. Untreated control groups were maintained in standard medium without extracts. The CCK-8 assay showed no significant differences in cell viability between the SpNWS-treated and control groups for both PC12 and L929 cells on Days 1, 3 or 5 (Fig. 3i and Fig. S20a). Live/dead staining similarly showed comparable cell morphology and density across all groups throughout the observation period (Fig. 3j and Fig. S20b). These findings indicate that the SpNWS does not induce detectable cytotoxicity.

The wireless stimulation efficacy of the SpNWS was experimentally confirmed in an acute rat sciatic nerve model. Following surgical implantation of the SpNWS, its efficacy was quantitatively evaluated through synchronized acquisition of gastrocnemius muscle tension and compound muscle action potential (CMAP) dynamics (Fig. 3k). Transdermal delivery of biphasic pulses (±5 V amplitude, 2 ms pulse width, single-cycle burst, 1 s pulse interval) generated phase-locked 1 Hz oscillations in both CMAP and muscle tension traces (Fig. 3l and m), indicating spatiotemporally precise neuromodulation of the sciatic nerve. To validate the anti-inflammatory efficacy of SpNWS-based SNS, an acute inflammation rat model was established via lipopolysaccharide (LPS) induction. Stimulation at amplitudes greater than ±5 V significantly alleviated organ damage and suppressed the increase in blood tumor necrosis factor-alpha (TNF-α) levels in the rats (Fig. S21). The *in vivo* biocompatibility of the PPGSA hydrogel electrodes within the SpNWS was further assessed by sciatic nerve implantation in mice for 10 days. Sham surgery groups underwent sciatic nerve isolation without implantation, while positive controls received nerve transection with suture ligation. The sciatic nerve in the implantation group showed no significant fibrous encapsulation, maintained intact neural architecture, and exhibited normal electrical signal transmission, demonstrating the excellent biocompatibility of the PPGSA electrode (Figs S22 and S23).

To accommodate the complex anatomical structure and fragile nature of the SNVBs, an adhesive hydrogel composed of polyacrylic acid and *N*-hydroxy succinimide-modified polyacrylic acid (PAA/PAA–NHS hydrogel) was applied to surface-modify the PPGSA hydrogel fiber electrodes to improve handling and minimize interfacial gaps that can lead to encapsulation. The modified electrodes showed strong adhesion to diverse tissues, including nerves, spleen and gastrocnemius muscle (Fig. S24). Mechanical testing confirmed that the adhesive hydrogel is significantly softer than PPGSA, suggesting it can act as a compliant interfacial buffer layer between the electrodes and SNVBs (Fig. S25a and b). Electrochemical impedance tests showed that the adhesive coating did not significantly affect the interfacial impedance of the PPGSA hydrogel, preserving its electrostimulation performance (Fig. S25c).

Effective electrical insulation is essential for neural interfaces to prevent unintended stimulation of surrounding healthy tissues and to enhance stimulation selectivity. The self-healing PUE was selected as the insulating layer due to its autonomous structural recovery and favorable biocompatibility [60,61]. Molecular engineering using polytetramethylene ether glycol (PTMEG) as soft segments and butanedione dioxime as chain extenders yielded PUE films with tissue-compatible mechanics, exhibiting kPa-level Young’s modulus comparable to soft biological tissues (Figs S26 and S27). Adhesion tests showed that PUE films rapidly formed strong interfaces, reaching interfacial energy (212.13 ± 29.49 J cm^−2^) and adhesion strength values (18.43 ± 1.10 kPa), respectively (Fig. S28). These results confirm the strong self-adhesive properties of PUE and its suitability as an insulating layer for neural interfaces on the SNVBs. The combination of tissue-adhesive PPGSA hydrogel fiber electrodes and instant self-adhesive PUE insulation enabled safe and convenient implantation of SpNWSs onto rats’ SNVBs (Fig. S29).

**Figure 4.**
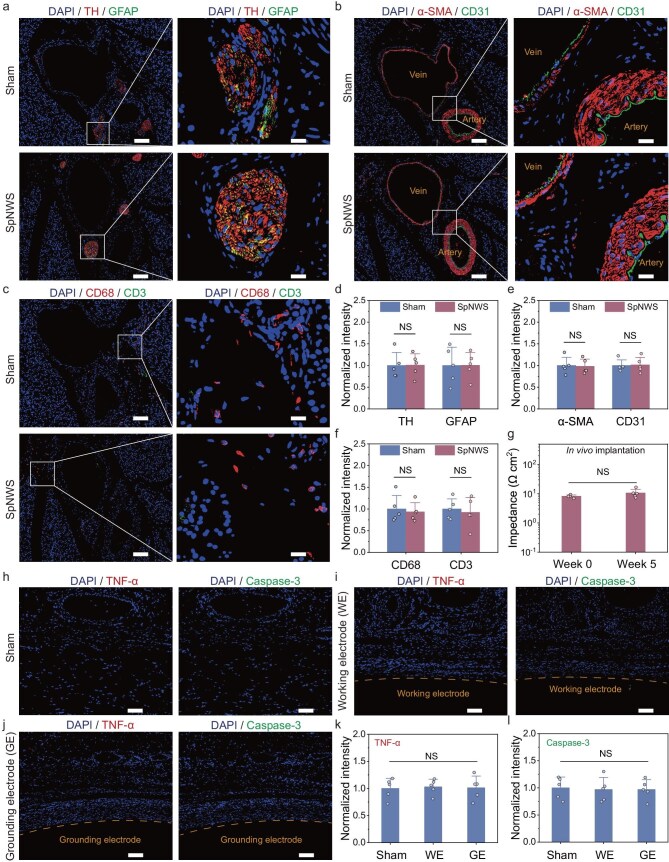
Long-term biocompatibility and stability of the SpNWS. (a–c) Representative immunofluorescence staining of SNVBs in the SpNWS and sham groups at 5 weeks post-implantation: (a) splenic nerves (TH) and astrocytes (GFAP), (b) fibroblasts (α-SMA) and endothelial cells (CD31), (c) macrophages (CD68) and T cells (CD3). Staining of the CD31 and α-SMA confirmed the positions of the splenic artery and vein. DAPI (4′,6-diamidino-2-phenylindole), a blue-fluorescent DNA stain, labels nuclei. Scale bars, 100 μm (left panels). Scale bars, 20 μm (right panels). (d–f) Normalized fluorescence intensity of SNVBs in the SpNWS and sham groups at 5 weeks post-implantation: (d) TH and GFAP, (e) α-SMA and CD31, (f) CD68 and CD3. The SpNWS group exhibited levels of neuroinflammation, fibrotic progression and immune responses in SNVBs comparable to the sham group (*n* = 5 independent animals). (g) Comparison of electrochemical impedance at 1 kHz for PPGSA electrodes before implantation and at 5 weeks post-implantation. The PPGSA electrodes showed no statistically significant reduction in electrochemical impedance (*n* = 5 independent animals). (h–j) Representative immunofluorescence staining of abdominal skin tissues in the SpNWS and sham groups at 5 weeks post-implantation: (h) sham group, (i) skin tissues against the working electrode of SpNWS, (j) skin tissues against the grounding electrode of SpNWS. TNF-α and caspase-3 serve as markers for inflammatory response and cellular apoptosis, respectively. Scale bars, 100 μm. (k and l) Normalized fluorescence intensity of abdominal skin tissues in the sham and SpNWS groups at 5 weeks post-implantation: (k) TNF-α, (l) caspase-3. The skin tissues against the working and grounding electrodes of the SpNWS exhibited levels of inflammatory response and cellular apoptosis comparable to the sham group (*n* = 5 independent animals). Data are presented as mean ± standard deviation in (d), (e), (f), (g), (k) and (l), and were analyzed by one-way ANOVA first, and then by the Tukey’s *post hoc* test. NS, not significant. (d) *P*_TH_ = 0.9685, *P*_GFAP_ = 0.9876. (e) *P*_α-SMA_ = 0.8649, *P*_CD31_ = 0.8946. (f) *P*_CD68_ = 0.6975, *P*_CD3_ = 0.6832. (g) *P*_week0 vs__._  _week5_ = 0.1907. (k) *P*_Sham vs__._  _WE_ = 0.9639, *P*_Sham vs__._  _GE_ = 0.9938. (l) *P*_Sham vs__._  _WE_ = 0.9679, *P*_Sham vs__._  _GE_ = 0.9686.

Immunofluorescence staining and hematoxylin and eosin (H&E) staining were performed 35 days after implantation to evaluate the long-term biocompatibility of the SpNWS. Rats that underwent the identical surgical procedure but without device implantation served as the sham group. No significant differences in body weight and major organ histology were observed between SpNWS and sham groups, indicating the absence of systemic toxicity (Figs S30 and S31). Tyrosine hydroxylase (TH) and glial fibrillary acidic protein (GFAP) were used to label splenic nerves and astrocytes, respectively (Fig. 4a). To characterize fibrosis and vascular damage at the implantation site, α-smooth muscle actin (α-SMA) and cluster of differentiation 31 (CD31) were employed to label myofibroblasts and vascular endothelial cells, respectively (Fig. 4b). CD3 and CD68 were used to assess immune cell infiltration by marking T cells and macrophages, respectively (Fig. 4c). No significant differences in TH and GFAP expression were observed between SpNWS and sham groups, indicating preserved neural integrity after long-term implantation (Fig. 4d). α-SMA and CD31 expression in the SpNWS group remained comparable to the sham group, suggesting no fibrosis or vascular disruption (Fig. 4e). These outcomes may be attributed to minimized interfacial gaps provided by the adhesive coating. H&E staining at the implantation site confirmed intact vascular architecture and neural structures in the SpNWS group (Fig. S32). CD68 and CD3 staining showed no increase in immune cell infiltration, indicating minimal chronic inflammation (Fig. 4f). Furthermore, impedance measurements of PPGSA electrodes remained stable over 5 weeks, confirming a reliable electrode–tissue interface (Fig. 4g).

The power harvesting modules of the SpNWS were subcutaneously implanted in the abdominal region of rats. Immunofluorescence staining of TNF-α and cysteine-dependent aspartate-specific protease-3 (caspase-3) was employed to analyze inflammatory responses and cellular apoptosis in skin tissue contacting the implanted modules (Fig. 4h–j). Although thin fibrous encapsulation formed around the working and grounding electrodes following chronic implantation, the expression levels of TNF-α and caspase-3 showed no statistically significant differences compared with the sham group, suggesting that prolonged electrode-tissue interfacing did not elicit detectable local immune activation or dermal injury (Fig. 4k, l and Fig. S33).

### Electroceutical treatment of IBD using an SpNWS

**Figure 5.**
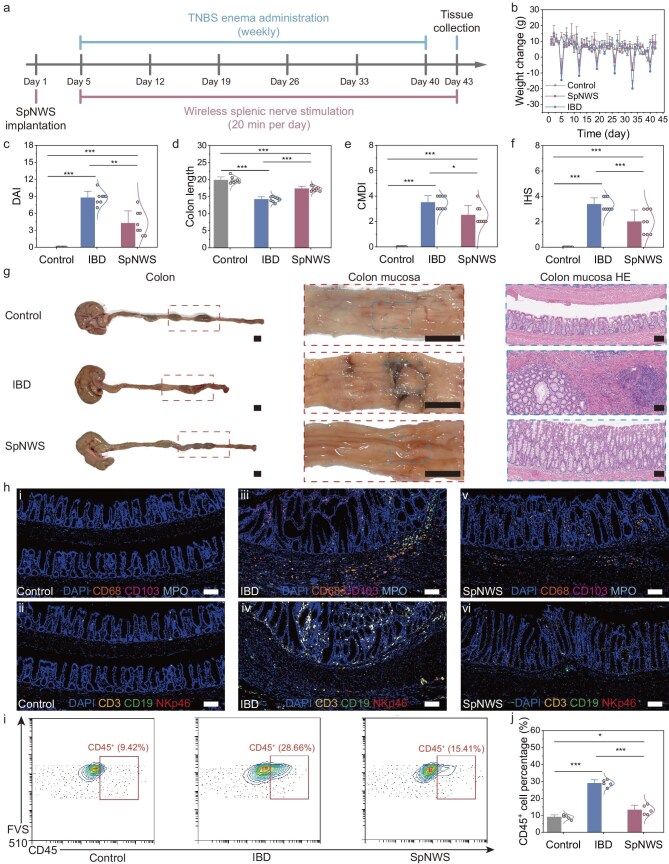
Electroceutical treatment of IBD with chronic SNS enabled by an SpNWS. (a) Schematic diagram of the experimental timeline showing key timepoints. The rat IBD model was established by TNBS enema. Electrical stimulation was initiated on Day 5 post-SpNWS implantation and maintained throughout the modeling period. (b) Body weight changes over time in the control, IBD and SpNWS groups. The SpNWS group exhibited less body weight loss than the IBD group after each TNBS enema administration (*n* = 8 independent animals). (c–f) Comparison of DAI (c), colon length (d), CMDI (e) and IHS (f) among the control, IBD and SpNWS groups. The SpNWS group had a longer colon length and lower DAI, CMDI and IHS scores compared with the IBD group (*n* = 8 independent animals). (g) Representative photographs of the colon, colonic mucosa and H&E-stained colonic sections in the control, IBD and SpNWS groups. Scale bars: 1 cm (photographs), 100 μm (H&E images). (h) Representative immunofluorescence staining of colonic tissues from the control, IBD and SpNWS groups: macrophages (CD68), mucosal dendritic cells (CD103), neutrophils (MPO), T cells (CD3), B cells (CD19) and NK cells (NKp46). Scale bars, 100 μm. (i) Flow cytometric analysis of CD45^+^ cells in colonic tissues from the control, IBD and SpNWS groups. CD45 is a pan-leukocyte marker expressed on all nucleated hematopoietic cells, including lymphocytes (T, B and NK cells), monocytes, dendritic cells and granulocytes. (j) Quantitative comparison of CD45^+^ cells in colonic tissues from the control, IBD and SpNWS groups. The SpNWS group exhibited fewer leukocytes than the IBD group (*n* = 5 independent animals). Data are presented as mean ± standard deviation in (a), (c), (d), (e), (f) and (j), and were analyzed by one-way ANOVA first, and then by the Tukey’s *post hoc* test. *P ≤ 0.05, **P ≤ 0.01, ***P ≤ 0.001. (c) *P*_SpNWS vs__._  _IBD_ = 8.8849 × 10^−6^. (d) *P*_SpNWS vs__._  _IBD_ = 3.7434 × 10^−7^. (e) *P*_SpNWS vs__._  _IBD_ = 0.0033. (f) *P*_SpNWS vs__._  _IBD_ = 5.6599 × 10^−4^. (j) *P*_SpNWS vs__._  _IBD_ = 1.4448 × 10^−8^.

IBD represents a typical refractory chronic condition characterized by chronic inflammation. While both VNS and sacral nerve stimulation have recently demonstrated therapeutic potential [34,62], SNS offers a more favorable therapeutic paradigm for refractory IBD due to its superior targeting specificity, most direct pathway, and clearly elucidated mechanism of action. Building upon the demonstrated stable neural interface and superior *in vivo* biosafety of the chronically implanted SpNWS, we next systematically investigated the therapeutic potential of SpNWS in a rat model of chronic IBD. Three experimental groups were established, comprising the control, IBD and SpNWS groups. The chronic IBD rat model was established through weekly 2,4,6-trinitrobenzenesulfonic acid (TNBS) enema administrations for 7 consecutive weeks [63]. The SpNWS was implanted on SNVBs 5 days before initial TNBS exposure, with daily 20-min stimulation (±5 V amplitude, 2 ms pulse width, single-cycle burst, 1 s pulse interval) initiated after the first enema (Fig. 5a). During electrical stimulation, the external power transmitter maintained precise alignment with the subcutaneously implanted working electrode, while the external grounding electrode remained aligned with the implanted grounding electrode (Fig. S34a and b). Throughout the 20-min stimulation period, no detectable thermal effects were observed in the capacitive coupling region (temperature elevation ∼1°C), confirming the operational safety in the SpNWS group (Fig. S34c).

To evaluate the therapeutic efficacy of the SpNWS intervention, body weight and fecal condition were monitored throughout the treatment period in the control, IBD and SpNWS groups. At the completion of the treatment, colon length, colonic mucosal injury and colonic inflammatory status were systematically assessed to comprehensively evaluate intestinal pathology and treatment response. Due to rapid growth in the rats, the control group maintained sustained weight gain, whereas the IBD and SpNWS groups exhibited pronounced weight loss immediately following each TNBS instillation due to colonic injury. However, this weight loss was significantly alleviated in the SpNWS group compared with the IBD group (Fig. 5b and Fig. S35). The disease activity index (DAI) score, which integrates body weight changes, stool consistency and fecal occult blood, was systematically applied to quantify disease progression across all groups. Compared with the control group, the IBD group showed higher DAI scores, reflecting weight loss, watery stools and bloody stool. In contrast, the SpNWS group had milder symptoms and lower DAI scores than the IBD group (Fig. 5c).

Colon shortening, a hallmark of IBD, was most pronounced in the IBD group compared with the control group, whereas electroceutical treatment restored colonic length toward normal levels in the SpNWS group (Fig. 5d). The colon mucosal damage index (CMDI) was scored based on macroscopic examination of post-treatment mucosal tissues to assess colonic damage severity. The IBD group displayed severe ulceration with necrotic tissue in colonic mucosa, accompanied by markedly elevated CMDI scores compared with the control group. In contrast, the SpNWS group’s colonic mucosa closely resembled that of the control group, with only mild ulceration, and showed significantly lower CMDI scores than the IBD group (Fig. 5e). The inflammation-related histology score (IHS) was assessed using post-treatment H&E-stained colonic sections to reflect inflammatory infiltration. The IBD group exhibited transmural immune cell infiltration with concomitant goblet cell depletion, resulting in significantly elevated IHS relative to the control group, while the SpNWS group displayed only focal inflammatory cell clusters and IHS values approaching physiological baselines (Fig. 5f and Fig. S36). Collectively, the SpNWS group showed significant alleviation of IBD symptoms compared with the IBD group, as indicated by body weight trajectories, gross intestinal morphology and histological analysis (Fig. 5g).

To further investigate colonic immune alterations induced by splenic nerve neuromodulation in IBD treatment, we employed immunofluorescence and flow cytometry to analyze immune cell populations, subtypes and spatial distribution in the control, IBD and SpNWS groups. Immunofluorescence staining utilized specific markers: CD3 (T cells), CD19 (B cells), natural killer cell p46-related protein (NKp46, NK cells), CD68 (macrophages), CD103 (dendritic cells) and myeloperoxidase (MPO, neutrophils) to precisely identify infiltrating immune cell subsets. In the control group, minimal infiltration by adaptive immune cells (T and B cells) was observed, whereas innate immune cells (macrophages, neutrophils, NK cells) were predominantly localized to the mucosa and lamina propria (Fig. 5h_i_ and h_ii_). In contrast, the IBD group displayed dense lymphoid aggregates enriched with T and B cells extending from the mucosa to submucosa, while NK cells, macrophages, neutrophils and dendritic cells showed transmural distribution (mucosa to muscularis) (Fig. 5h_iii_ and h_iv_). Critically, T cells extensively infiltrated mucosal and lamina propria regions in IBD rats, suggesting their pivotal role in pathogenesis (Fig. 5h_iv_). Importantly, splenic nerve neuromodulation in the SpNWS group significantly attenuated immune cell infiltration across all colonic layers compared with the IBD group (Fig. 5h_v_, h_vi_ and Fig. S37). Additionally, flow cytometry revealed reduced frequencies of CD45^+^ leukocytes in the SpNWS group, consistent with immunofluorescence staining results (Fig. 5i and j). Collectively, these findings support splenic nerve neuromodulation as an effective strategy to mitigate IBD-associated inflammation by reducing leukocyte recruitment.

### Electroceutical modulation of intestinal immune homeostasis in IBD

To investigate how SNS modulates intestinal immunity, we performed RNA sequencing on colonic tissues from the control, IBD and SpNWS groups. Five biological replicates per group underwent principal component analysis (PCA), with three high-concordance replicates selected for subsequent analysis (Fig. S38a). Venn diagram analysis identified 1594 differentially expressed genes (DEGs) common to both IBD vs. control and SpNWS vs. IBD comparisons (Fig. S38b and c). Among these DEGs, the IBD group exhibited upregulation of pro-inflammatory genes related to calprotectin (*S100a9*), chemokine networks (*Cxcl6*) and IL-1 signaling (*Il1rn*) compared with the control group (Fig. S39a). After SNS treatment, the SpNWS group showed significant suppression of these pro-inflammatory mediators and elevated expression of anti-inflammatory regulators (*Foxp3, Tgfb1, Pparg*), suggesting the therapeutic efficacy of splenic neuromodulation (Fig. S39b). Enrichment analysis showed marked upregulation of pro-inflammatory pathways (NF-κB, IL-17, TNF) in the IBD group, whereas the SpNWS group suppressed these pathways and enhanced anti-inflammatory signaling (PPARγ pathway) (Fig. S40). Furthermore, neuromodulatory pathways (neuroactive ligand–receptor interaction, serotonergic synapse and cholinergic synapse) were significantly enhanced by SNS (Fig. S40).

**Figure 6.**
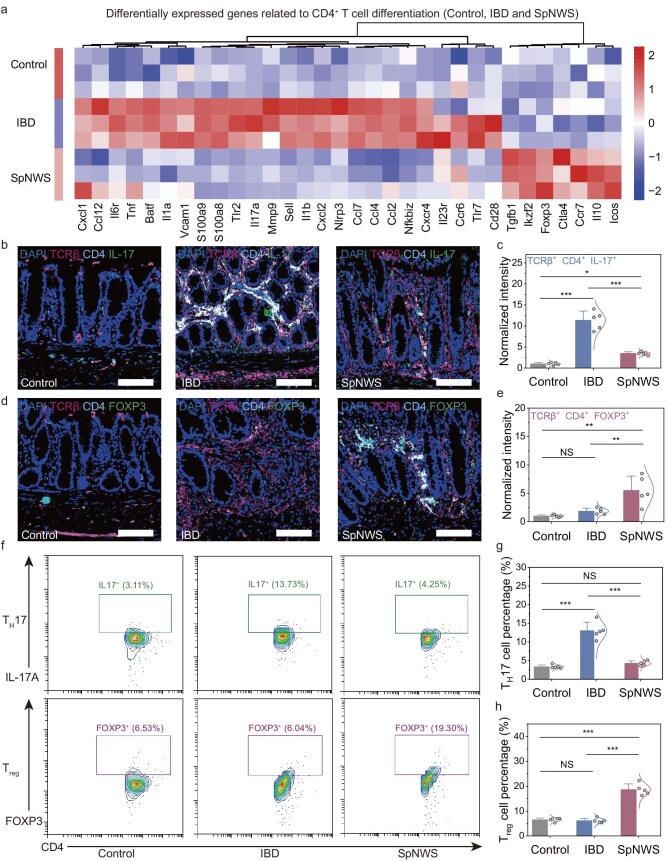
Colonic CD4^+^ T cell homeostasis restoration via SpNWS-enabled electroceutical therapy in IBD. (a) Hierarchically clustered heatmap of CD4^+^ T cell differentiation-associated DEGs in colonic tissues from the control, IBD and SpNWS groups. The majority of SpNWS-upregulated CD4^+^ T cell-associated genes were linked to T_reg_ cells, including *Foxp3, Tgfb1* and *Ctla4*. Conversely, those upregulated in the IBD group primarily correlated with T_H_1/T_H_17-type inflammatory responses, exemplified by *Tnf, Nfkbiz, Ccr6* and *Il17a*. (b) Representative immunofluorescence staining of T_H_17 cells in colonic tissues from the control, IBD and SpNWS groups. Cells triple-positive for TCRβ, CD4 and IL-17 are defined as T_H_17 cells. Scale bars, 100 μm. (c) Normalized fluorescence intensity of T_H_17 cells in colonic tissues from the control, IBD and SpNWS groups. T_H_17 cell populations were significantly reduced in the SpNWS group compared with the IBD group (*n* = 5 independent animals). (d) Representative immunofluorescence staining of T_reg_ cells in colonic tissues from the control, IBD and SpNWS groups. Cells triple-positive for TCRβ, CD4 and FOXP3 are defined as T_reg_ cells. Scale bars, 100 μm. (e) Normalized fluorescence intensity of T_reg_ cells in colonic tissues from the control, IBD and SpNWS groups. T_reg_ cell populations were significantly increased in the SpNWS group compared with the IBD group (*n* = 5 independent animals). (f–h) Fluorescence-activated cell sorting (FACS) plots (f) and quantitative comparisons of colonic T_H_17 (g) and T_reg_ (h) cell proportions among the control, IBD and SpNWS groups. The SpNWS group showed significantly decreased T_H_17 cell proportions and elevated T_reg_ cell frequencies compared with the IBD group (*n* = 5 independent animals). Data are presented as mean ± standard deviation in (c), (e), (g) and (h), and were analyzed by one-way ANOVA first, and then by the Tukey’s *post hoc* test. NS, not significant,*P ≤ 0.05, **P ≤ 0.01, ***P ≤ 0.001. (c) *P*_IBD vs__._  _SpNWS_ = 1.4525 × 10^−6^. (e) *P*_IBD vs__._  _SpNWS_ = 0.0056. (g) *P*_IBD vs__._  _SpNWS_ = 1.2436 × 10^−6^. (h) *P*_IBD vs__._  _SpNWS_ = 4.8156 × 10^−8^.

Given the established mechanistic link between CD4^+^ T cell dynamics and IBD disease development [64–66], we screened 32 genes associated with CD4^+^ T cell differentiation from the DEGs pool. The SpNWS group displayed specific upregulation of key regulators governing regulatory T (T_reg_) cell development, functionality, stability, and migration, exemplified by *Foxp3, Tgfb1*, and *Ctla4*. Conversely, the IBD group exhibited specific upregulation of genes enriched in inflammation/immune response pathways, particularly in those mediated by T helper cell 1 (T_H_1)/T_H_17-driven inflammatory cascades, including *Tnf, Nfkbiz, Ccr6* and *Il17a* (Fig. 6a). Therefore, SNS mediates immunomodulation through dual mechanisms: enhancing neuromodulatory pathways and anti-inflammatory signaling while suppressing pro-inflammatory networks, and these molecular mechanisms suggested potential regulatory effects on CD4^+^ T cell differentiation.

To further investigate SNS-mediated effects on CD4^+^ T cell differentiation in colitis, immunofluorescence and flow cytometric analyses were performed to quantify the proportions of T_H_1, T_H_2, T_H_17 and T_reg_ cells in colonic tissues from the control, IBD and SpNWS groups. CD4^+^ T cells were identified by coexpression of CD4 and the T cell receptor beta chain (TCRβ), with subsets distinguished via intracellular staining for interferon-gamma (IFN-γ, T_H_1), IL-4 (T_H_2), IL-17 (T_H_17), and forkhead box protein P3 (FOXP3, T_reg_). Immunofluorescence staining revealed significantly higher proportions of T_H_1 and T_H_17 cells in the IBD group versus the control group, whereas T_H_2 and T_reg_ cell proportions showed no significant change, indicating CD4^+^ T cell imbalance (Fig. 6b–e and Fig. S41). In the SpNWS group, T_H_1 and T_H_17 cell proportions were intermediate (significantly higher than the control group but lower than the IBD group), whereas T_H_2 and T_reg_ cell proportions were significantly elevated compared to both the control and IBD groups (Fig. 6b–e and Fig. S41).

Flow cytometric analysis, which employed enzymatic digestion of whole intestinal tissues for immune cell isolation coupled with rigorous gating strategies, provided more precise quantification of immune cell subsets compared with semi-quantitative immunofluorescence analysis (Fig. S42). Critically, flow cytometric results corroborated the immunofluorescence findings: T_H_1 and T_H_17 cell proportions were highest in the IBD group and significantly reduced following chronic SNS in the SpNWS group, conversely, T_H_2 and T_reg_ cell proportions exhibited a significant increase specifically in the SpNWS group (Fig. 6f–h and Fig. S43). These findings indicate that SpNWS-enabled SNS promotes the restoration of CD4^+^ T cell homeostasis in TNBS-induced IBD rats by shifting the immune profile toward dominance of anti-inflammatory T_H_2/T_reg_ cells while suppressing pro-inflammatory T_H_1/T_H_17 responses. This suggests that SpNWS-enabled SNS may constitute a novel therapeutic strategy for rebalancing immune dysregulation in colitis.

## CONCLUSION

In our previous experiments, we observed that implanting PDMS-based cuff electrodes into the SNVBs of rats and delivering wired stimulation via transcutaneous metal leads to the external stimulators resulted in high mortality rates. In this contribution, we developed a fully implantable hydrogel-based wireless neurostimulator with excellent mechanical compliance and biocompatibility, allowing long-term and robust electrical stimulation of splenic nerve. The SpNWS enables chronic SNS through the integration of wireless hydrogel bioelectronics specifically engineered for the anatomical constraints of SNVBs. By using a highly conductive and mechanically compliant PPGSA hydrogel, synthesized through the GSA strategy, the SpNWS forms stable neural interfaces without the need for mechanical fixation or sutures. GSA-induced polymer rearrangement promotes PEDOT:PSS phase separation and PVA crystallization, yielding a hydrogel with conductivity exceeding 3000 S m^−1^ and a Young’s modulus closely matched to splenic tissues. The fiber-shaped architecture conforms to irregular nerve geometries, while surface bioadhesive coatings allow for secure placement through wrapping and knotting. In a chronic IBD model, SpNWS-enabled SNS mitigated colitis symptoms, preserved mucosal structure and rebalanced CD4⁺ T cell subsets. These outcomes demonstrate that SpNWSs provide a fully implantable and robust neural interface capable of achieving effective and selective neuroimmune modulation.

The functional performance of the SpNWS remained stable during long-term implantation, with no signs of fibrotic encapsulation or immune activation at the tissue interface. Material properties can be precisely adjusted through hydrogel processing, allowing independent control of conductivity and mechanical compliance. Similarly, stimulation parameters including amplitude, frequency and pulse duration are tunable through the external capacitive input, enabling fine control over output characteristic without compromising device flexibility. Adjusting pulse width allows temporal control of stimulation, which may help improve specificity and minimize off-target activation. The combined tunability of material and electrical parameters supports the development of personalized stimulation strategies tailored to target-specific physiological responses, while maintaining compatibility with soft tissue and wireless power delivery.

The efficacy of SpNWSs is likely influenced by the dielectric and structural features of the surrounding tissue environment. Local factors such as hydration, vascular architecture and immune status may affect polarization dynamics and stimulation efficiency. Although the current study focused on SNS for treating IBD, the modular design and mechanical compliance of SpNWSs suggest broad applicability across other peripheral targets involved in inflammation, metabolism or autonomic regulation. Future studies should investigate how tissue-specific impedance profiles and anatomical variations shape neuromodulation effects and device performance. By combining soft tissue integration with chronic stability and wireless operation, SpNWSs establish a generalizable platform for next-generation bioelectronic therapies aimed at restoring systemic immune homeostasis through precision peripheral neuromodulation.

## METHODS

### Establishment of IBD model

Six-week-old SPF-grade male Sprague Dawley rats were housed in a GB14925-2010 standard barrier environment with sterilized feed and purified water regularly replaced. Three days of individual adaptive feeding were implemented before surgery. A TNBS-induced chronic IBD rat model was established through weekly TNBS enema administration (2.5% TNBS in 50% ethanol administered 8 cm from the anus into the colon at 50 mg kg^−1^) for six consecutive weeks. After each enema administration, rats were immediately positioned inverted for 2 min to minimize TNBS reflux. Body weight, fecal characteristic and other health indicators were monitored throughout the experiment. Terminal dissection was conducted on the third day after the final intervention to assess colonic macro-lesions, mesenteric lymph node status and mucosal changes, followed by H&E and immunofluorescence staining.

### Implantation of the SpNWS for SNS

Six-week-old SD rats were acclimatized for 3 days and fasted for 12 h preoperatively to minimize gastrointestinal interference during implantation. Surgical procedures were performed under isoflurane gas anesthesia. The left abdominal surgical area was shaved and disinfected sequentially with alcohol and iodophor. An incision was made through the skin and muscle layers, followed by gentle exteriorization of the spleen to expose the splenic hilar connective tissue and vascular bundles. The SNVBs were meticulously dissected, while the adhesive PPGSA conductive fibers of the SpNWS were looped around them and knotted to establish neuro–electrode interfaces. The interfaces were encapsulated with the biocompatible PUE thin-film for fixation and electrical insulation. The spleen was repositioned intra-abdominally, and muscle layers were sutured. Blunt dissection between the abdominal skin and muscle facilitated implantation of the working and grounding electrodes in the SpNWS, followed by skin closure. It should be noted that the non-insulated area of the grounding electrode in the SpNWS must be oriented facing externally during implantation to facilitate connection with the external grounding electrode for grounding during stimulation.

### Chronic SNS using the SpNWS

In the SpNWS group, SNS was delivered through biphasic charge-balanced rectangular pulses (±5 V amplitude, 2 ms pulse width, single-cycle burst, 1 s pulse interval) for 20 min daily. This regimen was initiated 5 days post-implantation and maintained throughout the modeling period. Stimulation procedures were conducted under isoflurane anesthesia administered via an RWD R583S rodent anesthesia system.

### Statistical analysis

The statistical analysis was conducted with OriginPro 2023 (Version 9.8.5.204) by one-way ANOVA first, and then by the Tukey’s *post hoc* test. Data are presented as mean ± standard deviation. The significance threshold was presented as * for *P* ≤ 0.05, ** for *P* ≤ 0.01 and *** for *P* ≤ 0.001, respectively; NS, not significant.

### Ethical statements

All animal experiments strictly followed the guidelines approved by the Institutional Animal Care and Use Committee of Huazhong University of Science and Technology (approval number: 4632).

## Supplementary Material

nwaf557_Supplemental_File
