# Supplementary material for: Chronic neurostimulation of splenic nerve enabled by hydrogel-bioelectronics for wireless electroceutical immunomodulation therapy
Source: Natl Sci Rev. 2025 Dec 5;13(2):nwaf557. doi: 10.1093/nsr/nwaf557 (PMC12831029; doi:10.1093/nsr/nwaf557)
Supplement: nwaf557_Supplemental_File [file nwaf557_supplemental_file.pdf]

Supplementary Information

Chronic neurostimulation of splenic nerve enabled by hydrogel-bioelectronics for wireless electroceutical immunomodulation therapy

Wenliang Liu[1,†], Qiong Wang[2,†], Renyuan Sun[1,†], Ming Yang[1,†], Ping Wu[3], Dingke Zhang[1], Kun Yang[1], Chong Ma[1], Chuan Gao[1], Nanxi Yi[1], Zhikun Li[1], Long Wen[2], Luyao Wu[2], Xiaokun Li[3], Jiexiong Feng[2,4,*], Zhouguang Wang[3,*] and Zhiqiang Luo[1,2,*]

[1]National Engineering Research Center for Nanomedicine, Research Center for Intelligent Fiber Devices and Equipment, State Key Laboratory of New Textile Materials and Advanced Processing, College of Life Science and Technology, Huazhong University of Science and Technology, Wuhan 430074, China;

[2]Department of Pediatric Surgery, Tongji Hospital, Tongji Medical College, Huazhong University of Science and Technology, Wuhan 430030, China;

[3]The First Affiliated Hospital of Wenzhou Medical University, Wenzhou 325035, China;

[4]Hubei Clinical Center of Hirschsprung's disease and allied disorders, Wuhan 430074, China

***Corresponding authors.** E-mails: 2002tj0515@hust.edu.cn; wangzhouguang@wmu.edu.cn; zhiqiangluo@hust.edu.cn

[†]Equally contributed to this work.

Supplementary Materials and Methods

Materials

Polyvinyl alcohol (PVA, Mw = 146,000-186,000, 99+% hydrolyzed) was purchased from Sigma-Aldrich, Inc. Glutaraldehyde (GA, 50wt% solution in H_2O), α-ketoglutaric acid (KA, 99%), and 1-butyl-3-methylimidazolium tetrafluoroborate ([BMIM]BF_4, 99%) were purchased from J&K Scientific. Dimethyl sulfoxide (DMSO, AR) was purchased from Shanghai Hushi Chemical Reagent Co., Ltd. Poly(3,4-ethylenedioxythiophene):poly(styrenesulfonate) (PEDOT:PSS, 1.1-1.3wt%) was purchased from Heraeus Electronic Materials. Glycerol (GL, Pharmaceutical grade) was purchased from Hunan Er-Kang Pharmaceutical Co., Ltd. Poly(tetrahydrofuran) (PTMEG, average Mn ~1000), N-N-dimethylacetamide (DMAC, ≥ 99.9%), isophorone diisocyanate (IPDI, mixture of isomers, 99%), dibutyltin dilaurate (DBTDL, 95%), dimethylglyoxime (DMG, 98%), triethanolamine (TEA, AR), acrylic acid (AA, ≥ 99.0%), and N-succinimidyl acrylate (AA-NHS, ≥ 98%) were purchased from Shanghai Aladdin Biochemical Technology Co., Ltd. Poly(ethylene glycol) diacrylate (PEGDA, average Mw ~700) was purchased from Shanghai Macklin Biochemical Co., Ltd. SYLGARD™ 184 Silicone Elastomer Kit was purchased from Dow Inc. Picrylsulfonic acid solution (TNBS, 5%) was purchased from Meilun Biotechnology Co., Ltd. DI water was collected from a Milli-Q water purification system (Millipore). All reagents were used without purification.

Preparation of PPGSA hydrogel

Polyvinyl alcohol (PVA, 3%) was dissolved in ~1% PEDOT:PSS solution at 90°C. After cooling to room temperature, glutaraldehyde (GA, 0.1%) was added, mixed thoroughly, and rapidly centrifuged for 30 s (5,000 r/min) to remove bubbles, yielding a solution containing

PVA, PEDOT:PSS, and GA. This solution was injected into silicone or polylactic acid (PLA) molds of specific shapes. After 6-hour room-temperature crosslinking, the molds were frozen overnight at -20°C and thawed at room temperature the next day (three freeze-thaw cycles total), then soaked repeatedly in copious deionized water three times at 37°C to remove residual GA, yielding PPG hydrogel. The PPG hydrogel was immersed in [BMIM]BF_4/DMSO solution (IL:DMSO = 1:2) for 6 hours at room temperature. After immersion, the hydrogel was washed six times with copious deionized water at 37°C to eliminate excess IL and DMSO, resulting in PPGS hydrogel. The PPGS hydrogel was soaked in glycerol overnight to replace water content. Subsequently, the glycerol-infused PPGS hydrogel was annealed at 130°C for 10 min, followed by six cycles of deionized water immersion at 37°C to remove residual GA, ultimately producing PPGSA hydrogel. The first and final washing solutions from each step of the PPGSA preparation process were analyzed using a Shimadzu UV-1750 ultraviolet-visible (UV-Vis) spectrophotometer.

Construction of SpNWS

Using transparent silicone tubing with an inner diameter of 0.2 mm as a mold, the PPGSA hydrogel fibers were fabricated following the PPGSA hydrogel preparation protocol. The central portion of PPGSA hydrogel fibers was encapsulated with PDMS (base:curing agent = 10:1, w/w), retaining approximately 2.5 cm unencapsulated fiber at both ends. One free end was secured with 0.3 mm transparent silicone tubing, supplemented with PAA/PAA-NHS hydrogel precursor solution (~65% H_2O, ~35% AA, 0.1% AA-NHS, 0.1% PEG-DA, 0.2% KA), and UV-cured in a chamber (354 nm, 12 W power) for 30 minutes to surface-modify with

adhesive PAA/PAA-NHS hydrogel. At the opposite end, the working electrode component was connected to a 1 cm diameter circular PPGSA hydrogel film fully encapsulated with PDMS; the grounding electrode component was attached to a 8 mm × 3 mm rectangular PPGSA hydrogel film partially encapsulated with PDMS while retaining an uninsulated area of approximately 6 mm^2. It is noteworthy that before PDMS encapsulation, the PPGSA hydrogel fibers and films were appropriately dried at room temperature for approximately 10 minutes, resulting in reduced size and the formation of irregular wrinkles on the hydrogel surface to enhance the stability between PDMS and hydrogel. Both electrode components of the SpNWS were hermetically sealed with hydrated cotton balls for moisture retention and sterilized via UV irradiation prior to use.

Physicochemical characterization of PPGSA hydrogel

The three lyophilized hydrogel films (PPG, PPGS, and PPGSA hydrogels) were cryofractured in liquid nitrogen, and their cross-sectional morphologies were subsequently examined by scanning electron microscopy (SEM, SU8010, Hitachi, Japan). The same films were characterized using a confocal Raman spectrometer (LabRAM HR800, Horiba Jobin Yvon, France) with a 532 nm excitation wavelength. Additionally, the crystalline structures of the hydrogel films were analyzed by X-ray diffraction (XRD, X'Pert3 Powder, Malvern Panalytical, Netherlands). Structural information was further obtained via combined small- and wide-angle X-ray scattering (SAXS/WAXS, Xeuss 3.0 UHR, Xenocs, France). Surface chemical compositions of the hydrogel films were investigated by X-ray photoelectron spectroscopy (XPS, AXIS-ULTRA DLD, Kratos Analytical, Japan). Finally, the S 2p peaks

corresponding to the PSS and PEDOT components were semi-quantitatively evaluated using XPSPeak 4.1 software with Gaussian-Lorentzian peak fitting methodology.

Mechanical tests of PPGSA hydrogel

The tensile and compressive properties of PPG, PPGS, and PPGSA hydrogels were evaluated using a universal testing machine (CTM8000, Xie Qiang Instrument Manufacturing Co., Ltd., China). All hydrogels were equilibrated in 1× PBS through swelling prior to testing. For tensile characterization, rectangular specimens were fabricated using PLA molds with standardized dimensions: 30 mm (length) × 5 mm (width) × 2 mm (thickness). Uniaxial tensile testing was performed at a constant speed of 20 mm min^{-1}. For compressive assessments, cylindrical samples with 8 mm diameter and 4 mm height were prepared using PLA molds. Compression tests were conducted at 20 mm min^{-1} until reaching 50% strain. The elastic moduli were derived from the linear elastic region (about 20% strain) of stress-strain curves through linear regression analysis.

The rheological characterization of PPG, PPGS, and PPGSA hydrogels was performed using a rotational rheometer (MCR 102, Anton Paar, Austria) with temperature control maintained at 37°C. Cylindrical specimens measuring 8 mm diameter × 4 mm height were fabricated using PLA molds. Amplitude sweep tests (0.01%-100% strain) were first conducted at 1 Hz to establish the linear viscoelastic region (LVR). Subsequent frequency sweep measurements (0.5-10 rad s^{-1}) were then performed within the determined LVR at a constant strain amplitude of 0.05%. The complex shear modulus was calculated using Eq. (1):

$$|G^{*}| = \sqrt{G'^{2} + G''^{2}} \quad (1)$$

where G' and G'' represent the storage modulus and loss modulus at 1 Hz, respectively.

Electrical characterization of PPGSA hydrogel

The electrical conductivity of PPG, PPGS, and PPGSA hydrogels and the electrical resistance of the PPGSA hydrogel fiber under various strain conditions were characterized using an LCR meter (TH2830, Changzhou Tonghui Electronic Co., Ltd., China). Prior to characterization, all hydrogel specimens were equilibrated in 1× PBS through swelling equilibrium. The specimens, which had a rectangular geometry with precise dimensions of 20 mm in length, 20 mm in width, and 0.2 mm in thickness, were fabricated using PLA molds. The conductivity (σ) was calculated according to Eq. (2):

$$\sigma = \frac{L}{R \times W \times T} \tag{2}$$

where R represents the impedance magnitude measured at 1 kHz, with L, W, and T corresponding to the specimen's length, width, and thickness, respectively.

The electrochemical impedance spectroscopy (EIS) characteristics of PPG, PPGS, and PPGSA hydrogels were quantified utilizing a CHI660E electrochemical workstation (Shanghai Chenhua Instrument Co., Ltd., China). Cyclic voltammetric (CV) analysis and charge injection capacity (CIC) evaluations were performed on these hydrogel materials through a multichannel potentiostatic system (CHI1040C, Shanghai Chenhua Instrument Co., Ltd., China). All electrochemical characterizations were executed in 0.1 M PBS electrolyte employing a standardized three-electrode configuration. Fiber-shaped hydrogel specimens with geometric parameters of 0.3 mm diameter and 10 mm length were prepared for testing. Au fiber electrodes

with sizes similar to PPGSA hydrogel fibers were selected as control specimens. All hydrogel specimens were equilibrated in 1× PBS through swelling prior to testing.

EIS measurements were performed by applying a 5 mV sinusoidal voltage at 0 V DC bias across the frequency range of 0.1-100,000 Hz. CV analysis was conducted between -0.5 V and +0.5 V with a 50 mV s^{-1} scan rate. The charge storage capacity (CSC) was calculated using Eq. (3):

$$CSC = \int_{E_2}^{E_1} \frac{i(E)}{2vA} dE \tag{3}$$

where v denotes the scan rate, E_1 and E_2 define the potential window, i represents the potential-dependent current, and A corresponds to the electrode area.

The CIC was evaluated through application of biphasic voltage pulses (±0.5 V) at 50 Hz frequency. The parameter was subsequently calculated via Eq. (4):

$$CIC = \frac{Q_a + Q_c}{A} \tag{4}$$

where Q_a denotes the anode charge accumulation, Q_c represents the charge passed through the cathode, and A corresponds to the electrode area.

Wireless capacitive-coupling tests of SpNWS

Waveform editing was conducted using ArbExpress software (Tektronix, USA) to create a composite waveform containing a 1 MHz sine wave (0.5 V) combined with a biphasic charge-balanced rectangular pulse wave. The waveform was imported into a function generator (AFG3021C, Tektronix, USA) connected to a broadband amplifier (ATA-1200B, Aigtek, China). During testing, the input voltage was adjusted from 2.5 V to 8.5 V by modifying the amplifier's gain. The power-transmitter electrode was connected to the positive output of the

broadband amplifier, while the external grounding electrode was linked to the negative output. The external grounding electrode was aligned with the SpNWS neurostimulator's grounding electrode, and correspondingly, the power-transmitter electrode was aligned with the neurostimulator's working electrode. The neurostimulator's working electrode was connected to the digital oscilloscope's positive probe, with its grounding electrode connected to the ground reference.

During measurements, the digital oscilloscope displayed real-time open-circuit voltages of the coupling system across all input levels (2.5-8.5 V). For short-circuit current measurements, the current preamplifier's (SR570, Stanford Research Systems, USA) output was routed to the digital oscilloscope through a BNC interface. The power-transmitter electrode was interfaced with the broadband amplifier's output terminal, while the working electrode was connected to the current preamplifier's input. Real-time short-circuit currents of the coupling system were simultaneously monitored using the oscilloscope-current preamplifier assembly under varying excitation voltages (2.5-8.5 V).

For voltage and current measurements under varying loads, the testing protocol followed the aforementioned methodology with additional series-connected resistive loads (10 Ω to 10 MΩ) implemented at the working electrode. To evaluate the the coupling system's electrical outputs across different coupling distances, the separation between power-transmitter and working electrodes was systematically modulated. This spatial adjustment was implemented by interposing biological tissues with defined thicknesses, including 1-mm rat skin, 2-mm chest muscle, 3-mm composite muscle-skin layers, 10-mm gastrocnemius muscle, and 11-mm

integrated gastrocnemius muscle-skin specimens. The voltage transmission efficiency at varied misaligned distances, defined as the ratio of the induced voltage to the input voltage (5 V), was calculated from measurements taken at electrode misalignments of 0, 1, 2, 3, 4, and 5 mm.

After implanting the adhesive PPGSA conductive fiber of SpNWS neurostimulator around the splenic vascular wall and encapsulating the interface with biocompatible PUE film, we performed *in vivo* electrical characterization in isoflurane-anesthetized animals. Voltage across the splenic nerve was measured differentially by connecting an oscilloscope with high-input-impedance probes to two points on the nerve, allowing capture of amplitudes. The total current delivered by the stimulator was measured concurrently by inserting a current amplifier in series with the stimulator-lead-electrode-tissue loop.

Cytocompatibility assay of SpNWS

The cytocompatibility of SpNWS was evaluated using CCK-8 assay and live/dead cell staining with L929 (catalog: QS-M020, Keycell Biotechnology) and PC12 (catalog: QS-R009, Keycell Biotechnology) cell lines. SpNWS samples were extracted by immersion in Dulbecco's modified Eagle medium (DMEM, Solarbio) at 37°C for 24 h (100 mg mL^{-1}). Before testing, extracts were supplemented with 10% v/v fetal bovine serum (FBS, Umedium He Fei China) and 100 U mL^{-1} penicillin-streptomycin (PS, Solarbio). Cells were seeded in 24-well plates at densities of 3,000 (L929) and 2,000 (PC12) cells per well, then maintained at 37°C and 5% CO_2 for 1, 3, and 5 days. Viability was quantified using CCK-8 assay measured with a multi-mode microplate reader (TECAN Spark 10M, Switzerland). For morphological assessment, 10,000 (L929) and 6,000 (PC12) cells per well were cultured in 6-well plates under

identical conditions. Live/dead staining was analyzed using a FV3000 confocal microscope (Olympus, Japan).

Acute stimulation of sciatic nerve and splenic nerve using SpNWS

Electrophysiological signals were acquired using a biosignal acquisition system (BL-420N, Chengdu Tai Meng Software Co., Ltd.). The left sciatic nerve was surgically exposed and wrapped with the SpNWS neurostimulator's adhesive PPGSA hydrogel fiber electrodes. Needle electrodes were implanted in the left gastrocnemius muscle and connected to the system's input port. The capacitive coupling system's external device was attached to the skin surface using medical ultrasound coupling gel, aligned with the implanted neurostimulator. Electrical stimulation signals were delivered through the external device, with concurrent acquisition of gastrocnemius muscle action potentials via the implanted needle electrodes connected to the bioelectrical signal acquisition system. For tension recording, the left gastrocnemius tendon was isolated and connected to the system's force transducer via surgical sutures. Muscle tension changes were recorded during identical stimulation protocols.

The effectiveness of acute splenic nerve stimulation was validated using an acute inflammation model in rats. After implantation of the SpNWS, acute inflammation was induced by intravenous injection of lipopolysaccharide (LPS; Sigma-Aldrich, 10 mg/kg) via the femoral vein. Immediately following LPS injection, 20 minutes of wireless splenic nerve stimulation (2 ms pulse width, single-cycle burst, 1 s pulse interval) was applied. The experimental groups included: an LPS-only group (LPS injection without stimulation), a control group (no LPS and no stimulation), and three stimulation groups with different voltage amplitudes (5 V, 10 V, and

15 V). Blood samples were collected hourly for four hours via carotid artery catheterization, and plasma TNF-α concentrations were measured using a rat TNF-α ELISA kit (Sigma-Aldrich). After the experiment, the liver, lung, and kidney were harvested from each group for hematoxylin and eosin (H&E) staining.

Preparation and characterization of PUE films

The typical preparation procedure for PUE films is described below. PTMEG (14.5 g, 15 mmol) was added to a dried glass flask equipped with a mechanical stirrer. The system was dehydrated at 100°C under vacuum for 1 h, then cooled to 70°C under nitrogen atmosphere. A DMAc solution (5 mL) containing isophorone diisocyanate (6.77 g, 30.45 mmol) and DBTDL (0.05 g, 2000 ppm) was introduced via dropwise addition, followed by stirring at 70°C for 2 h to form the prepolymer. After cooling to room temperature, dimethylglyoxime (1.683 g, 14.5 mmol) dissolved in DMAc (10 mL) was added as chain extender, and the reaction proceeded for 12 h under sealed conditions. Subsequently, triethylamine (TEA, 80 μL, 0.603 mmol) was introduced, followed by an additional 6 h of reaction. The resulting mixture was concentrated to 30 wt% using DMAc (30 mL), yielding the PUE solution. For PUE films preparation, the 30 wt% PUE dispersion was diluted to 10% with DMSO. A 12 mL aliquot of the diluted dispersion was evenly cast in a polytetrafluoroethylene circular mold (diameter = 12 cm). The cast film was dried at 80°C to remove residual DMAc solvent, ultimately producing PUE films with controlled thickness of approximately 200 μm. The chemical structure of the PUE films was characterized by nuclear magnetic resonance spectroscopy (NMR, AV400, Bruker, Switzerland) and Fourier transform infrared spectroscopy (FTIR, Nicolet iS50R, USA).

Mechanical and self-healing tests of PUE films

The mechanical and self-healing properties of PUE films were evaluated using a universal testing machine (CTM8000, Xie Qiang Instrument Manufacturing Co., Ltd., China). For tensile testing, PUE specimens (30 mm × 5 mm × 2 mm) were prepared by casting 30% PUE solution into rectangular polytetrafluoroethylene molds followed by DMAc evaporation. Uniaxial tensile measurements were performed at a constant speed of 50 mm min^{-1}. Young's modulus was determined from the initial linear regime of the stress-strain curves. The self-healing capability of PUE films was assessed by pressing two PUE specimens (30 mm × 5 mm × 0.2 mm) under a plumb for 1 min at room temperature, followed by peeling and lap shear tests. Both tests were conducted at 20 mm min^{-1} after pressing. The interfacial toughness was calculated by dividing twice the peeling force by the overlap width, while the adhesive strength was determined by dividing the maximum shear force by the overlapping area.

Transcriptome sequencing

Upon completion of the SpNWS-based electroceutical IBD treatment, colonic tissues (from cecal terminus to ~1.5 cm anterior to anus) were collected from the control, IBD, and SpNWS groups. The colon tissues were rinsed with cold PBS to remove blood and residual tissues. Luminal contents were cleared through mesenteric-side incision and flushing. Tissues were sectioned into 1 cm pieces, flash-frozen in liquid nitrogen, and stored at -80°C. For sequencing, frozen samples were thawed and homogenized. Total RNA was extracted using TRIzol reagent (≥ 1 μg target concentration). RNA purity and integrity were respectively verified by NanoDrop ND-2000 and Agilent 4200 TapeStation. mRNA isolation from 1 μg RNA used oligo(dT) beads, followed by fragmentation and cDNA synthesis. Adapter ligation

and PCR amplification were conducted. Libraries were quantified (Qubit2.0 Fluorometer), sized (Agilent 4200 TapeStation), and concentration-validated (qRT-PCR). Normalized libraries were pooled and sequenced on an Illumina NovaSeq 6000. The sequencing mode was PE150.

Flow cytometry assay

The colonic tissues were initially rinsed in cold PBS to remove clots and adhesions. The tissues were sectioned into 0.3 cm segments, subjected to pre-digestion, and repeatedly filtered until mucus-free. These segments were subsequently minced, enzymatically digested, filtered, and rinsed with culture medium to generate single-cell suspensions. The remained tissues underwent mincing, filtration, mechanical grinding, and centrifugation for erythrocyte depletion before resuspension. Cell suspensions from the colonic tissues underwent viability staining with FVS510, FcγII/III receptor blockade, and surface marker labeling (CD45, CD4, TCR α/β) prior to flow cytometric analysis of $CD4^+$ T cells. For $T_H1/T_H2/T_H17/T_{reg}$ detection, single-cell suspensions were stimulated for 6 hours with leukocyte activation cocktail in a humidified 37°C and CO_2 incubator before FVS510 staining. Following surface marker labeling, cells underwent fixation/permeabilization with a transcription factor staining buffer kit prior to intracellular cytokine staining (IFN-γ, IL-4, IL-17A, FOXP3). Prepared samples were analyzed by the flow cytometer (CytoFLEX, Beckman Coulter, USA) using standardized acquisition parameters.

Histopathological and immunofluorescence examination

Following the SpNWS-based chronic electroceutical intervention, splenic neurovascular bundles, intestinal tissues, skin, heart, liver, spleen, lung, and kidney specimens from rats in

the control, IBD, and SpNWS groups underwent fixation in 4% paraformaldehyde with subsequent dehydration and paraffin embedding. Tissue sections were prepared for hematoxylin-eosin (H&E) staining to assess histopathological features. Immunofluorescence analyses included TH and GFAP staining for neural injury evaluation, α-SMA and CD31 detection for fibrosis and vascular damage characterization, and CD3 with CD68 labeling for immune response profiling at the neural interface. The extent of skin tissue injury and inflammatory response was assessed via dual immunofluorescence staining for Caspase-3 and TNF-α. The inflammatory status of intestinal tissues was systematically evaluated using multiplex immunofluorescence staining, with quantitative analysis of immune cell subsets achieved through specific labeling of macrophages (CD68), dendritic cells (CD103), neutrophils (MPO), T lymphocytes (CD3), B lymphocytes (CD19), and natural killer cells (NKp46). T cell subpopulations were identified using TCRβ and CD4 co-staining followed by IFN-γ, IL-4, IL-17A, and FOXP3 labeling to distinguish T_H1, T_H2, T_H17, and T_{reg} cells. Fluorescence intensity quantification was performed using ImageJ software.

Supporting figures

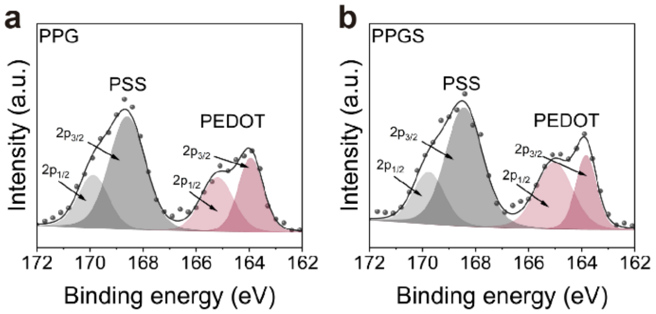

Figure S1. XPS analysis of PPG and PPGS hydrogels. (a, b) High-resolution S 2p XPS spectra of PPG (a) and PPGS (b) hydrogels, showing peak deconvolution for PSS and PEDOT components. All binding energies were calibrated to the C 1s peak at 284.8 eV.

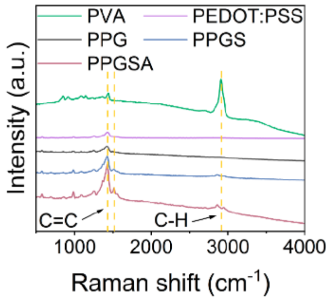

Figure S2. Raman characterization of PPG, PPGS, and PPGSA hydrogels. Raman spectroscopy of PPG, PPGS and PPGSA hydrogels reveals a progressive increase in the C=C (thiophene ring) vibration of PEDOT (1430 cm^{-1} and 1510 cm^{-1}) and the asymmetric CH stretching vibration of PVA (2910 cm^{-1}) during PPGSA hydrogel synthesis.

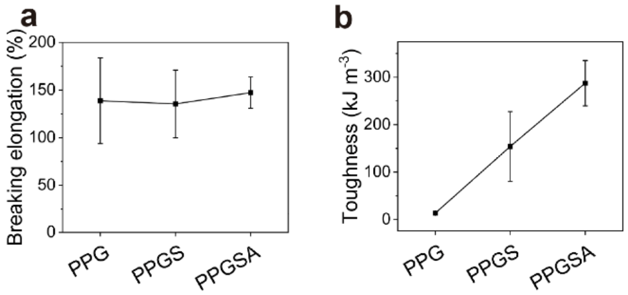

Figure S3. Breaking elongation and toughness of PPG, PPGS, and PPGSA hydrogels. (a) Breaking elongation of PPG, PPGS and PPGSA hydrogels, representing the ultimate tensile strain at material fracture (n = 3 independent hydrogel samples). (b) Tensile toughness calculated through stress-strain curve integration for PPG, PPGS and PPGSA hydrogels (n = 3 independent hydrogel samples). Data are presented as the mean ± standard deviation.

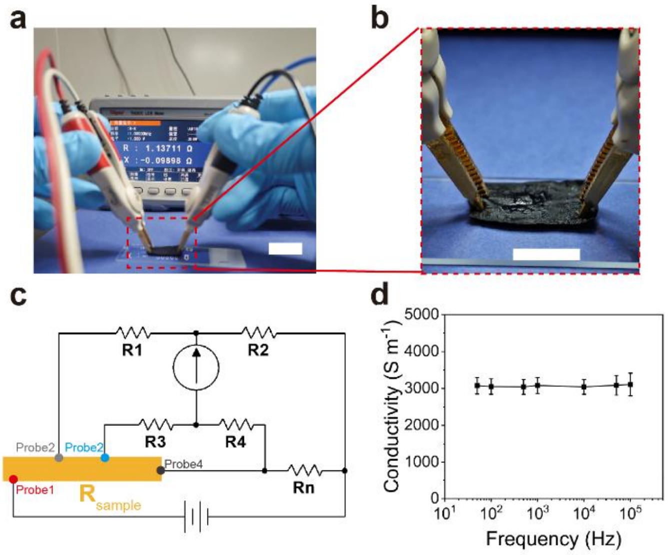

Figure S4. Electrical conductivity measurements of PPGSA hydrogel. (a) Photograph of PPGSA hydrogel conductivity measurements using a four-electrode bridge method with an LCR meter. Scale bar, 2 cm. (b) Magnified view showing probe-hydrogel contact interface in PPGSA hydrogel conductivity testing. Scale bar, 1 cm. (c) Equivalent circuit diagram for the conductivity measurements using a four-electrode bridge method. (d) Frequency-independent conductivity curve of PPGSA hydrogel measured by a four-electrode bridge method (n = 3 independent hydrogel samples). Data are presented as the mean ± standard deviation in (d).

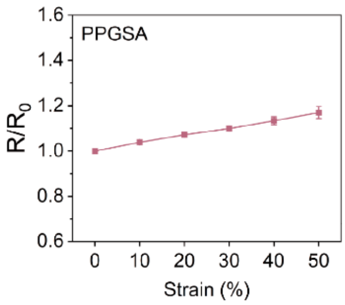

Figure S5. Resistance change rate (R/R_0) of PPGSA conductive hydrogel fibers under various tensile strains. The resistance of PPGSA hydrogel increases by less than 10% at 20% strain (n = 5 independent PPGSA hydrogel fibers).

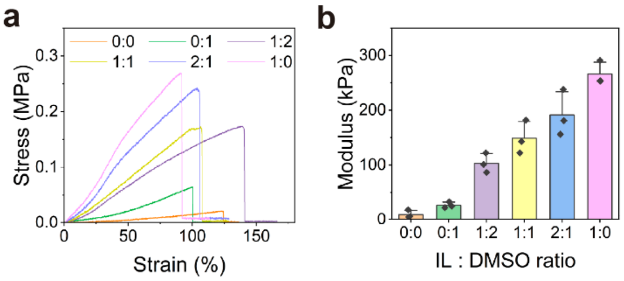

Figure S6. Tensile properties of PPGS hydrogels prepared with different IL/DMSO ratios. (a, b) Stress-strain curves (a) and Young's modulus (b) of PPGS hydrogels prepared with different [BMIM]BF_4/DMSO ratios (0:0, 0:1, 1:2, 1:1, 2:1, 1:0) (n = 3 independent hydrogel samples). Data are presented as the mean ± standard deviation in (b).

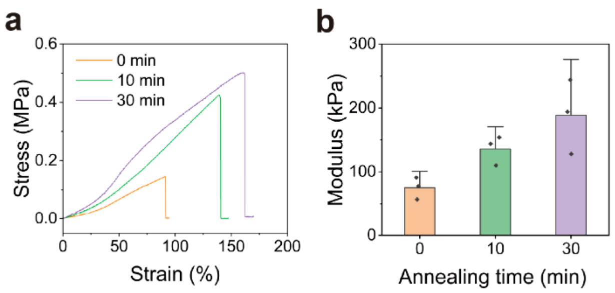

Figure S7. Tensile properties of PPGSA hydrogels prepared with different annealing times. (a, b) Stress-strain curves (a) and Young's modulus (b) of PPGSA hydrogels prepared with different annealing times (0, 10, 30 min) (n = 3 independent hydrogel samples). Data are presented as the mean ± standard deviation in (b).

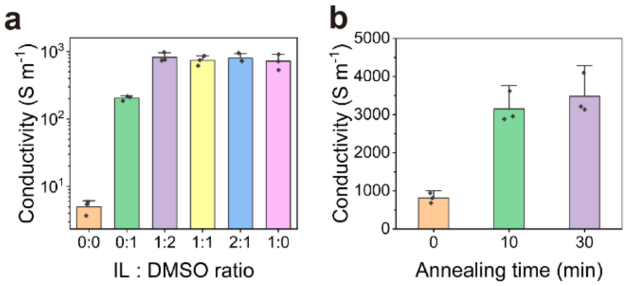

Figure S8. Conductivity of PPGS hydrogels prepared with different IL/DMSO ratios and PPGSA hydrogels fabricated with varying annealing times. (a) Conductivity variation of PPGS hydrogels with different [BMIM]BF_4/DMSO ratios (0:0, 0:1, 1:2, 1:1, 2:1, 1:0), showing enhanced conductivity with increased ionic liquid concentration reaching a plateau at 2:1 ratio (n = 3 independent hydrogel samples). (b) Conductivity evolution of PPGSA hydrogels under varied annealing durations (0, 10, 30 min), demonstrating progressive conductivity improvement with extended annealing time (n = 3 independent hydrogel samples). Data are presented as the mean ± standard deviation.

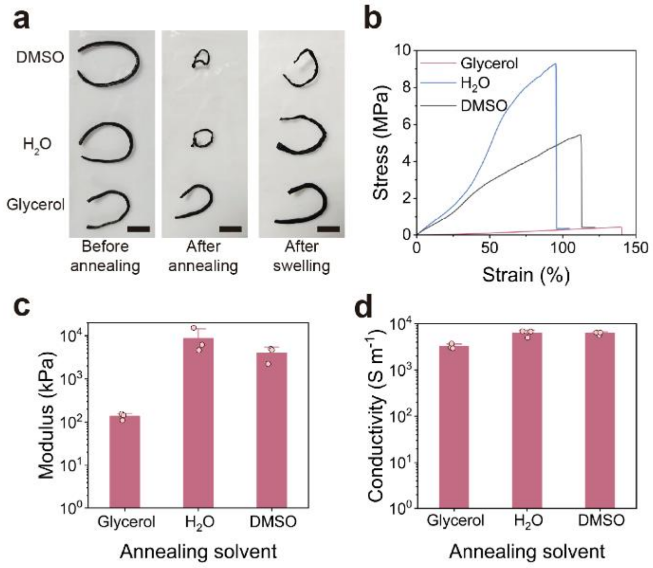

Figure S9. Tensile properties and electrical conductivity of PPGSA hydrogels prepared with different annealing solvents. (a) Photographs comparing PPGSA hydrogels prepared with DMSO, H_2O and glycerol as annealing solvents before and after annealing. Scale bar, 1 cm. (b-d) Stress-strain curves (b), Young's modulus (c), and conductivity (d) comparison of PPGSA hydrogels prepared with different anneling solvents (n = 3 independent hydrogel samples). Data are presented as the mean ± standard deviation in (c) and (d).

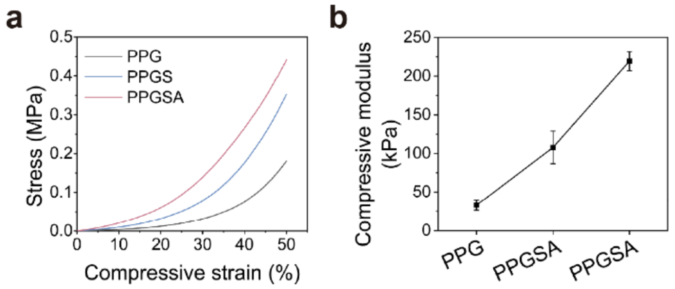

Figure S10. Compressive properties of PPG, PPGS, and PPGSA hydrogels. (a, b) Compressive stress-strain curves (a) and compressive modulus (b) of PPG, PPGS, and PPGSA hydrogels, showing continuously increasing compressive modulus during PPGSA hydrogel preparation reaching approximately 220 kPa (n = 3 independent hydrogel samples). Data are presented as the mean ± standard deviation in (b).

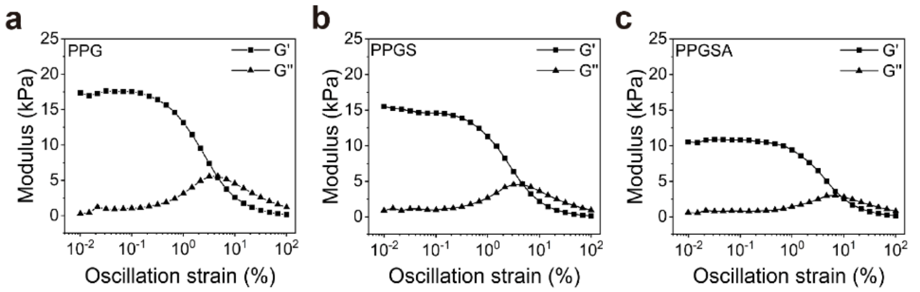

Figure S11. Oscillatory strain amplitude sweep of PPG, PPGS, and PPGSA hydrogels. (a-c) Oscillatory strain amplitude sweep (0.01-100%) rheological profiles of PPG (a), PPGS (b), and PPGSA (c), demonstrating maximum strain in linear viscoelastic region (LVR) of ~0.05%.

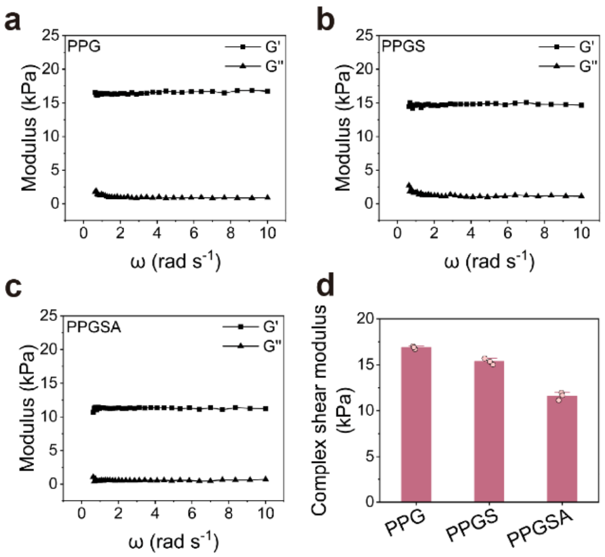

Figure S12. Oscillatory frequency sweep of PPG, PPGS, and PPGSA hydrogels. (a-c) Oscillatory frequency sweep (0.1-10 rad s^{-1}) rheological profiles of PPG (a), PPGS (b), and PPGSA (c). d Complex shear modulus of PPG, PPGS, and PPGSA hydrogels, with PPGSA hydrogel at approximately 12 kPa (n = 3 independent hydrogel samples). Data are presented as the mean ± standard deviation in (d).

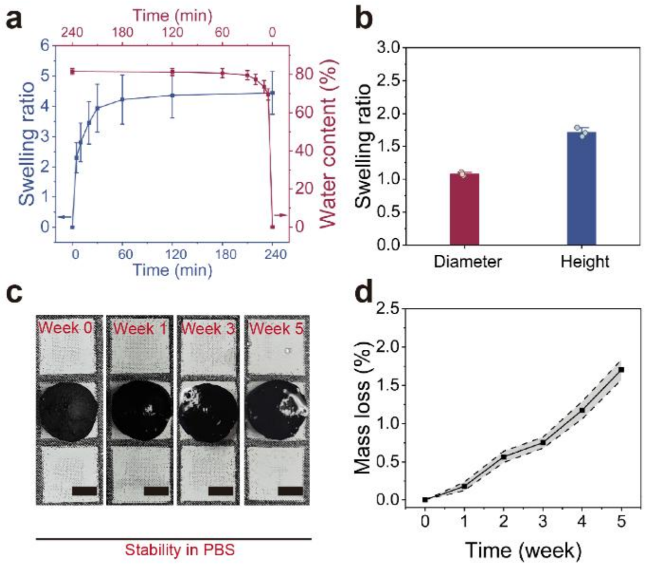

Figure S13. Swelling properties of PPGSA hydrogel. (a) Swelling ratio and water content curves of freeze-dried PPGSA hydrogel cylinders in PBS (37°C) over time, showing swelling equilibrium reached within ~2 h (n = 3 independent hydrogel samples). Swelling ratio is defined as the mass increase percentage relative to the initial state at each time point. Water content is calculated as the mass increment relative to the initial state divided by the current mass. (b) Post-swelling dimensional changes of freeze-dried PPGSA hydrogel in PBS (37°C) at equilibrium, demonstrating predominant axial swelling (>70% height increase) with minimal radial expansion (<10% diameter change) (n = 3 independent hydrogel samples). (c) Photographic record of PPGSA hydrogel samples in PBS (37°C) after 5-week immersion, indicating no significant dimensional alterations. Scale bar, 5 mm. (d) Mass loss profile of PPGSA hydrogel in PBS (37°C) during 5-week immersion, revealing <2% cumulative mass depletion (n = 3 independent hydrogel samples). Data are presented as the mean ± standard deviation in (a), (b), and (d).

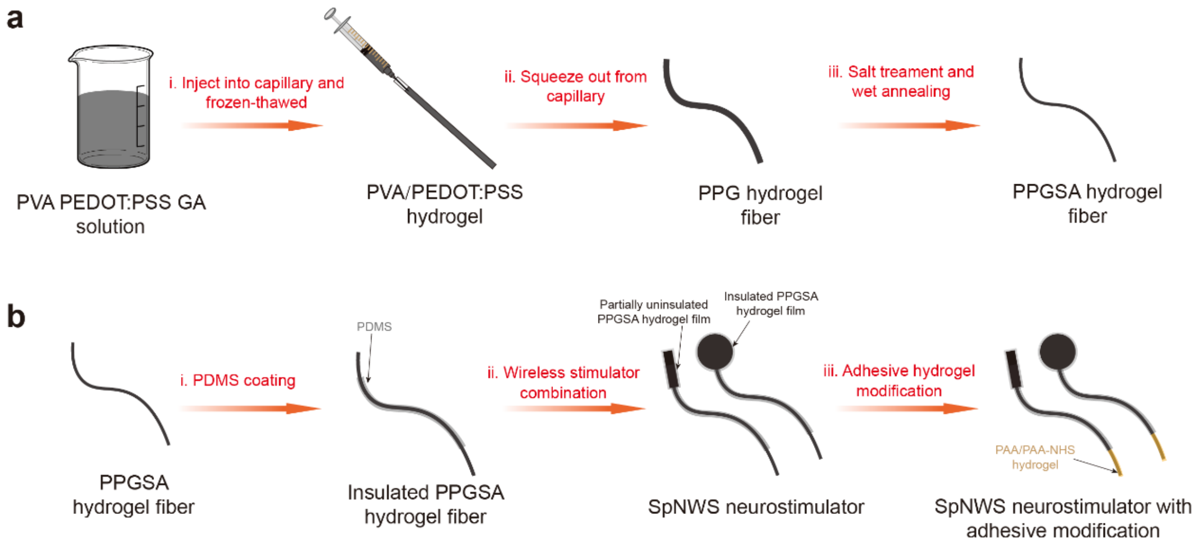

Figure S14. Schematic of PPGSA hydrogel fiber fabrication and SpNWS configuration. (a) Schematic illustration of PPGSA hydrogel fiber fabrication using silicone rubber tubing (inner diameter: 0.2 mm) as template combined with GSA strategy. (b) Construction schematic of SpNWS based on PPGSA hydrogel and wireless capacitive coupling technology, featuring insulation treatment of hydrogel fibers, integration of capacitive coupling receiver modules (PPGSA hydrogel films), and adhesive modification of hydrogel fiber electrodes.

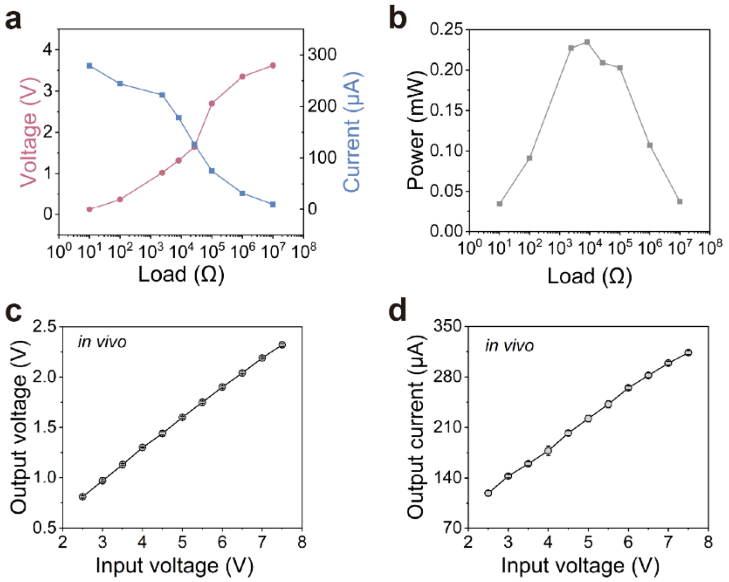

Figure S15. Capacitively coupled wireless transmission performance of SpNWS under varied loads. (a) Coupled voltage and current evolution of SpNWS under ±5 V input with varying loads (10^1-10^7 Ω), showing voltage increase and current decrease with rising load (n = 5 independent working electrodes). (b) Coupled power evolution of SpNWS under ±5 V input with varying loads (10^1-10^7 Ω), peaking within 2-10 kΩ range (~0.23 mW, calculated as voltage × current, n = 5 independent working electrodes). (c) Output voltages generated by the working electrode of the *in vivo* implanted SpNWS under varying input voltages (n = 5 independent SpNWS). (d) Output currents generated by the working electrode of the *in vivo* implanted SpNWS under varying input voltages (n = 5 independent SpNWS).

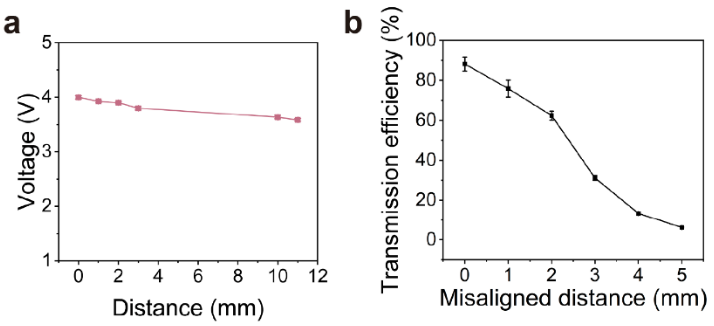

Figure S16. Capacitively coupled wireless transmission performance of SpNWS at varied coupling and misaligned distances. (a) Output voltage of SpNWS at varied coupling distances. As the coupling distance increases from 0 to 11 mm, the SpNWS under ±5 V input demonstrates less than 10% voltage reduction (n = 5 independent SpNWS samples). (b) Voltage transmission efficiency of SpNWS at varied misaligned distances (n = 5 independent SpNWS samples). Data are presented as the mean ± standard deviation.

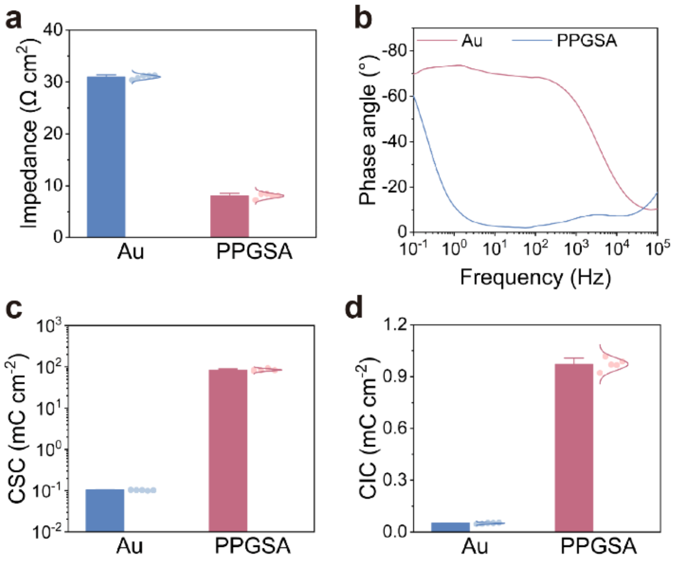

Figure S17. Electrochemical properties of PPG, PPGS, and PPGSA hydrogel fibers. (a) Electrochemical impedance comparison of PPGSA hydrogel fiber electrodes versus Au electrodes at 1 kHz. The electrochemical impedance of PPGSA hydrogel fiber electrodes measured approximately one-fourth that of Au electrodes (n = 5 independent PPGSA samples). (b) Phase angle spectra comparison of PPGSA hydrogel fiber electrodes versus Au electrodes. The PPGSA hydrogel fiber electrodes exhibited markedly lower phase angles than Au electrodes within the mid-frequency range (1 Hz-1 kHz). (c) CSC comparison of PPGSA hydrogel fiber electrodes versus Au electrodes. The CSC of PPGSA hydrogel fiber electrodes achieved approximately 800 times that of Au electrodes (n = 5 independent PPGSA samples). (d) CIC comparison of PPGSA hydrogel fiber electrodes versus Au electrodes. The CIC of PPGSA hydrogel fiber electrodes reached approximately 20 times that of Au electrodes (n = 5 independent PPGSA samples). Data are presented as the mean ± standard deviation in (a), (c), and (d).

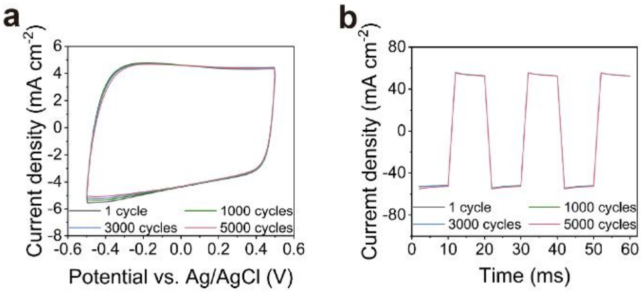

Figure S18. CSC and CIC stability of PPGSA hydrogel fibers. (a) Cyclic voltammetry (CV) stability profiles of PPGSA hydrogel fiber electrodes, demonstrating negligible variation over 5,000 cycles. (b) Cyclic charge injection capacity (CIC) retention of PPGSA hydrogel fiber electrodes, showing minimal signal decay through 5,000 cycles.

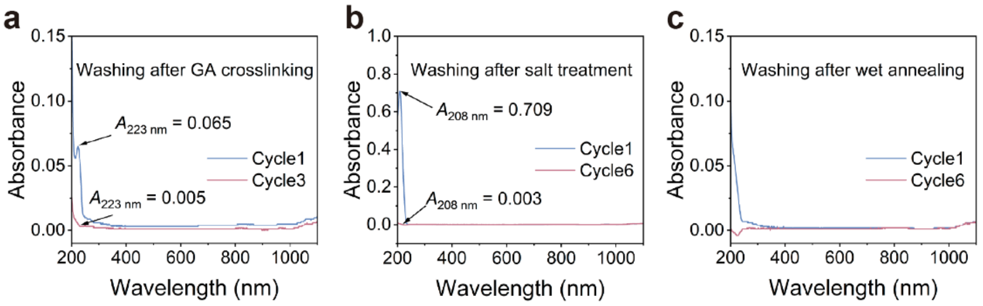

Figure S19. Effectiveness of the washing protocol in PPGSA hydrogel preparation. (a-c) UV-Vis spectra of washing solutions after the first and final washes following GA crosslinking (a), salt treatment (b) and wet annealing (c). The peak at 223 nm corresponds to the characteristic absorption of glutaraldehyde; the absorption at 208 nm represents the characteristic peaks of both DMSO and [BMIM]BF_4; while glycerol shows no distinct characteristic peak, it exhibits a continuously increasing absorption trend from 400 nm to 200 nm.

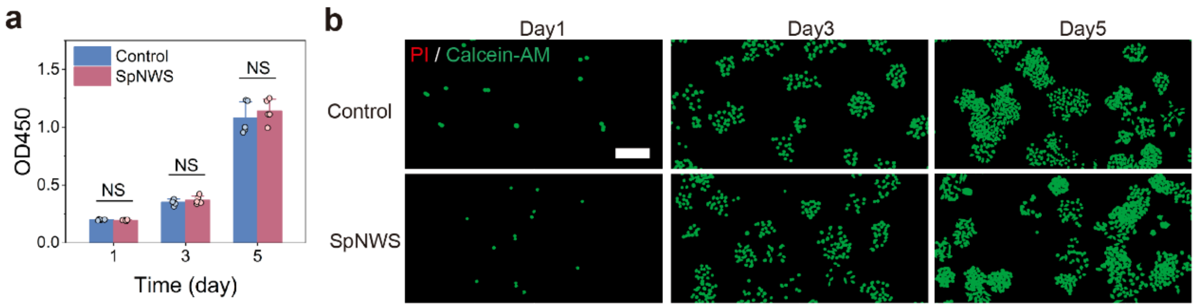

Figure S20. In vitro cytotoxicity assay of SpNWS. (a) Viability of L929 cells co-cultured with the SpNWS extract at 1, 3, and 5 days. L929 cells exposed to SpNWS extract demonstrated comparable proliferation kinetics to blank counterparts, with no statistically significant intergroup variation observed (n = 5 independent SpNWS samples). (b) Representative live-dead staining images of L929 cells co-cultured with the SpNWS extract at 1, 3, and 5 days. L929 cells demonstrated comparable cytomorphological characteristics and population density between the SpNWS extract-treated group and the blank group. Scale bar, 100 μm. Data are presented as the mean ± standard deviation in (a) and were analyzed by one-way ANOVA first, and then by the Tukey's post hoc test. NS, not significant. (a) $P_{day1} = 0.16057$, $P_{day3} = 0.41137$, $P_{day5} = 0.46236$.

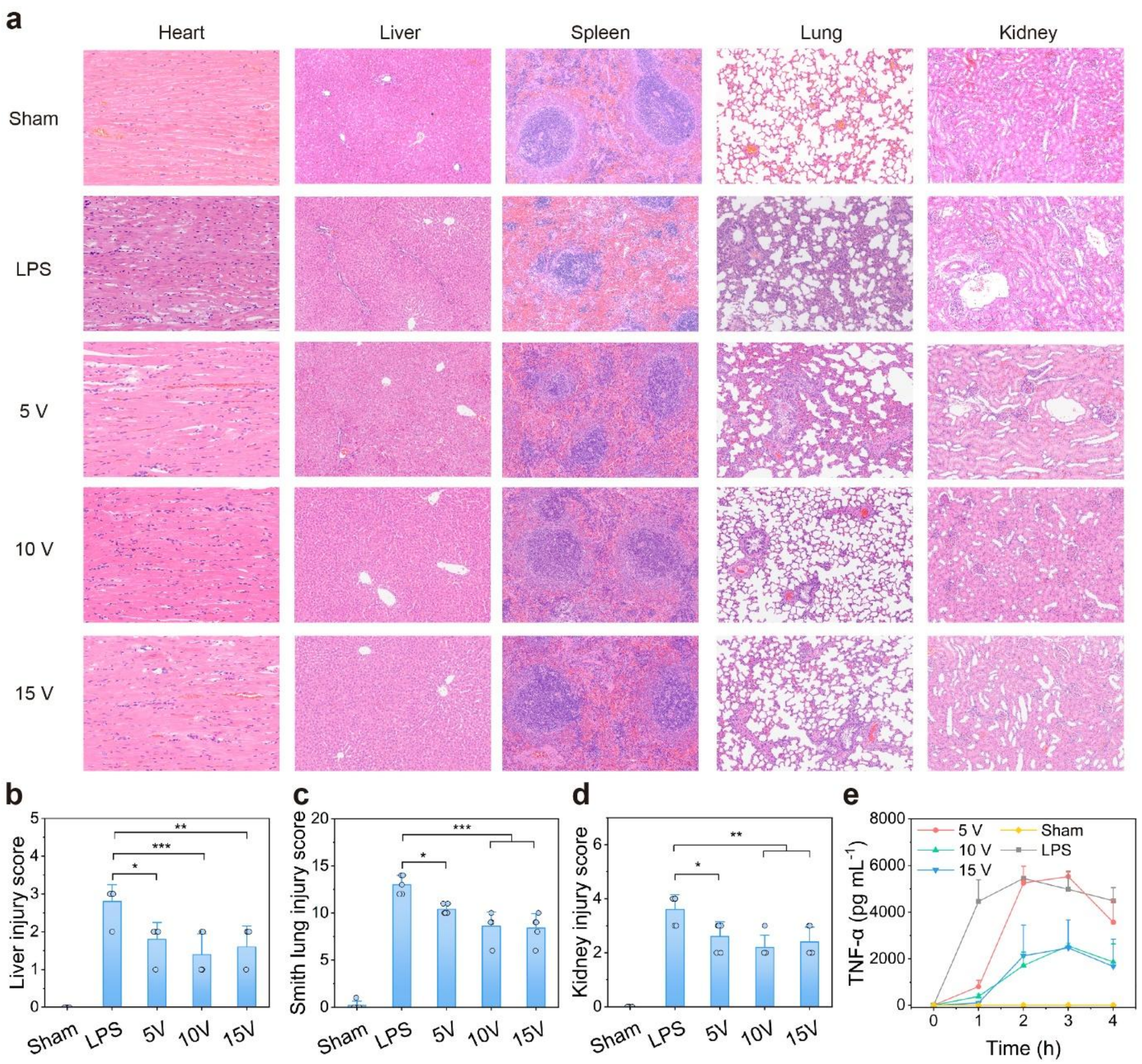

Figure S21. Anti-inflammatory effects of spleen nerve stimulation based on SpNWS in LPS-induced acute rat models. (a) H&E staining results of major organs (heart, liver, spleen, lung, kidney) in LPS-induced rats following splenic nerve stimulation at different intensities. (b-d) Injury scores for the liver (b), lung (c), and kidney (d) in LPS-induced rats after splenic nerve stimulation at different intensities (n = 5 independent animals). (e) Changes in the plasma level of the inflammatory cytokine TNF-α following splenic nerve stimulation at different intensities (n = 5 independent animals).

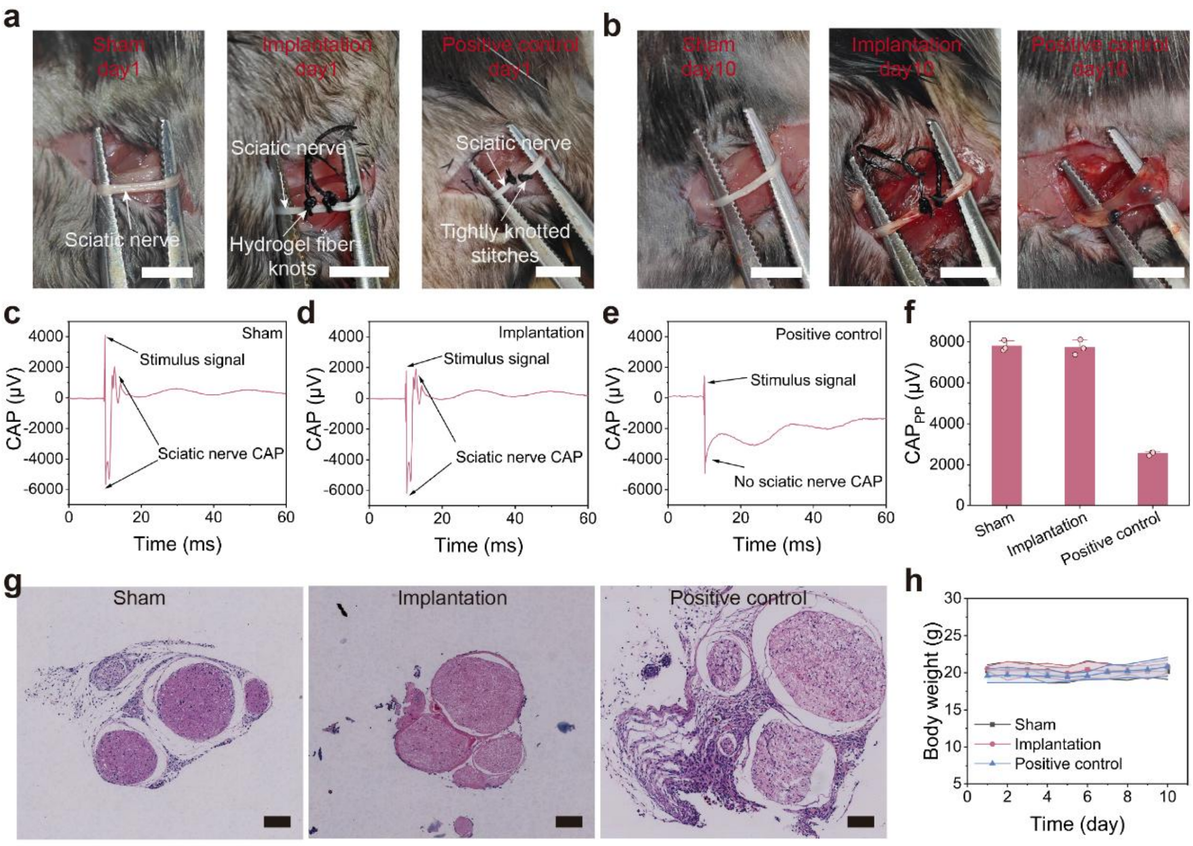

Figure S22. Neurophysiological architecture integrity of murine sciatic nerve following long-term implantation of PPGSA hydrogel fibers. (a, b) Images of sciatic nerves in sham, implantation, and positive control groups at day 1 (a) and day 10 (b). Scale bar, 5 mm. (c-e) Sciatic nerve compound action potential (CAP) recorded from sham (c), implantation (d), and positive control (e) groups under electrical stimulation (80 μA, 1 Hz) at day 10. (f) Quantitative sciatic nerve CAP peak-to-peak (CAP_{PP}) value comparison in sham, implantation, and positive control groups at day 10 (n = 3 independent animals). (g) H&E staining of sciatic nerves in sham, implantation, and positive control groups at day 10. Scale bar, 100 μm. (h) Body weight trajectories of the sham, implantation, and positive control groups over 10-day implantation period (n = 3 independent animals). Data in (f, h) are presented as the mean ± standard deviation.

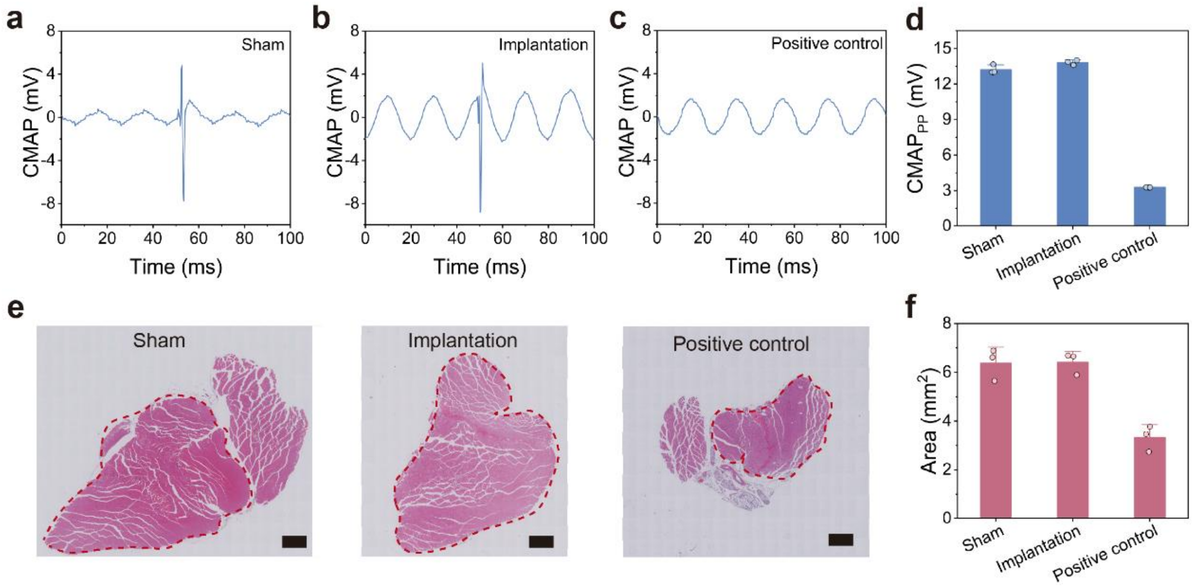

Figure S23. Neurological functionality safety assessment of murine sciatic nerve following long-term implantation of PPGSA hydrogel fibers. (a-c) Gastrocnemius compound muscle action potentials (CMAP) in the sham (a), implantation (b), and positive control (c) groups under sciatic nerve electrical stimulation (80 μA, 1 Hz) at day 10 post-operation. (d) Quantitative gastrocnemius CMAP peak-to-peak ($CMAP_{PP}$) value comparison in the sham, implantation, and positive control groups at postoperative day 10, demonstrating preserved signal magnitudes in implanted group (n=3 independent animals). (e) H&E staining of sciatic nerve-innervated gastrocnemius muscle crss-sections in the sham, implantation, and positive control groups at postoperative day 10. The area enclosed by the red dashed line indicates the maximum cross-sectional area of the gastrocnemius muscle. Scale bar, 500 μm. (f) Comparative analysis of maximum cross-sectional areas in sciatic nerve-innervated gastrocnemius muscles among the sham, implantation, and positive control groups at postoperative day 10. demonstrating absence of muscular atrophy in implantation group (n = 3 independent animals). Data in (d, f) are presented as the mean ± standard deviation.

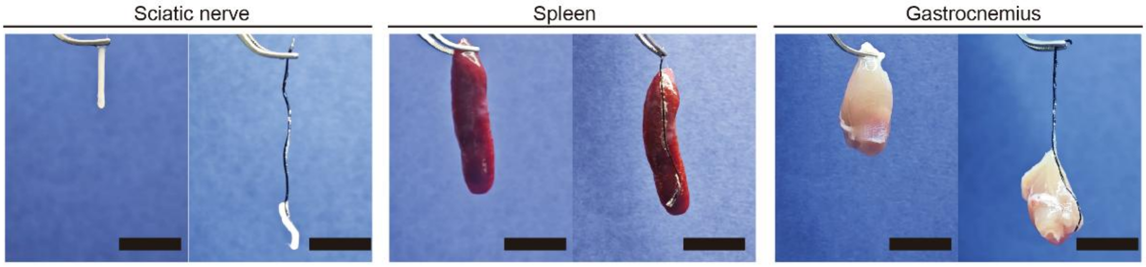

Figure S24. Bioadhesive performance of the PAA/PAA-NHS modified PPGSA hydrogel fibers. The PAA/PAA-NHS modified PPGSA hydrogel fibers exhibit the ability to adhere to and lift the rat sciatic nerve, spleen, and even the entire gastrocnemius muscle, thereby demonstrating superior tissue adhesion performance. Scale bar, 1 cm.

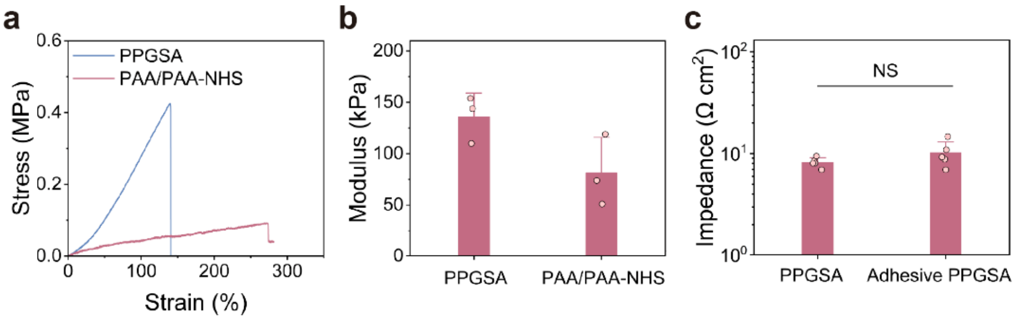

Figure S25. Tensile properties and electrochemical impedance of the PAA/PAA-NHS hydrogel. (a) Comparative stress-strain profiles of PAA/PAA-NHS and PPGSA hydrogels. (b) Young's modulus comparison between PAA/PAA-NHS and PPGSA hydrogels, demonstrating marginally lower Young's modulus in PAA/PAA-NHS hydrogel (~80 kPa) compared with PPGSA hydrogel (n=3 independent hydrogel samples). (c) Electrochemical impedance comparison of PPGSA hydrogel fiber electrodes and PAA/PAA-NHS modified PPGSA hydrogel fiber electrodes at 1 kHz, indicating that adhesive modification does not significantly increase the impedance of PPGSA hydrogels (n = 5 independent hydrogel samples). Data are presented as the mean ± standard deviation in (b) and (c). Data in (c) were analyzed by one-way ANOVA first, and then by the Tukey's post hoc test. NS, not significant. (c) $P_{\text{PPGSA vs Adhesive PPGSA}} = 0.19777$.

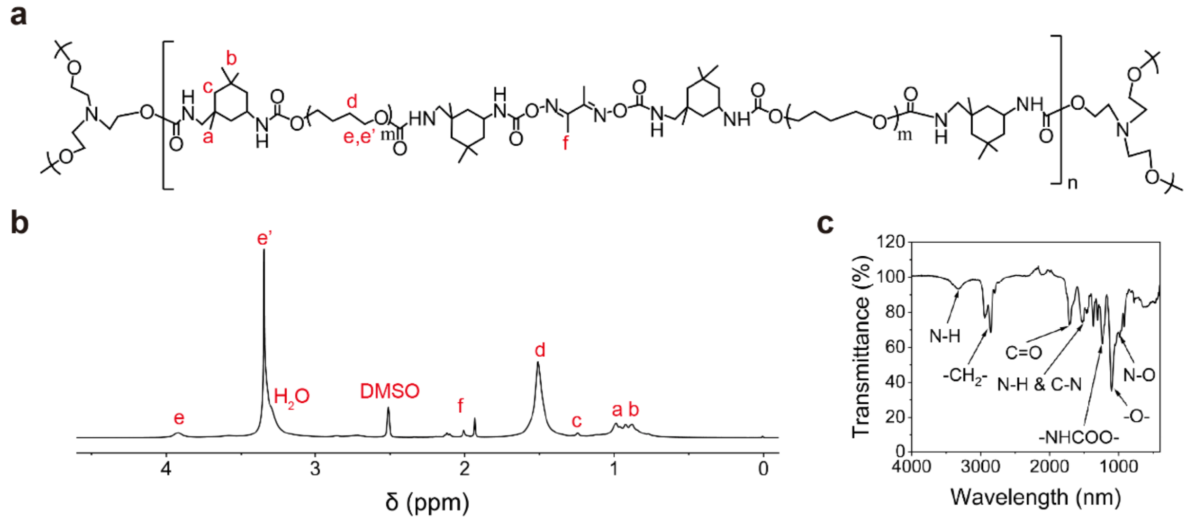

Figure S26. Structure characterization of the PUE. (a) Chemical structure of PUE with highlighted proton environments. (b) ^{1}H NMR spectrum of PUE showing characteristic proton peak assignments. (c) FT-IR spectral profile of PUE with key functional group annotations.

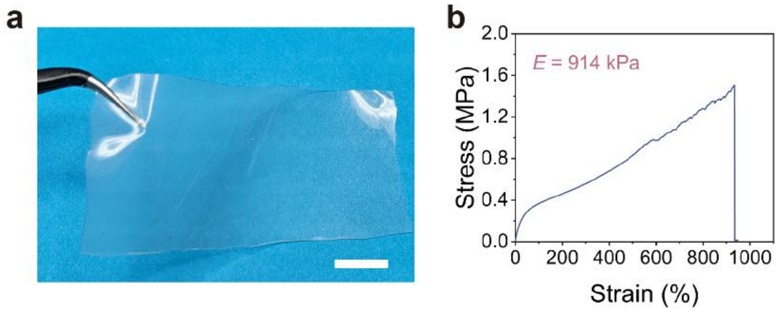

Figure S27. Tensile properties of the PUE. (a) Photograph of a PUE film with a thickness of ~200 μm. Scale bar, 1 cm. (b) Tensile stress-strain curve of PUE demonstrating a Young's modulus of ~914 kPa (n = 3 independent PUE samples).

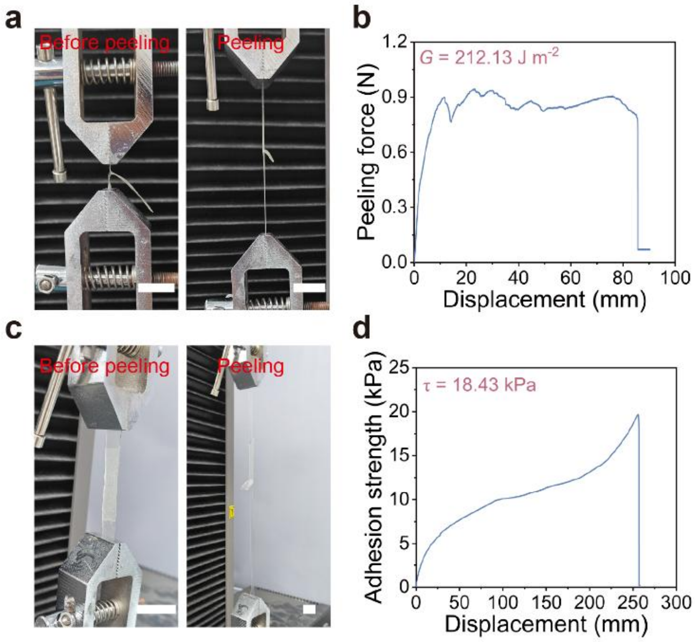

Figure S28. Self-healing properties of the PUE. (a) Images of two self-healed PUE films before and after peeling in T-peel tests. Scale bar, 2 cm. (b) A peeling force-displacement curve of two self-healed PUE films with interfacial energy of 212.13 ± 29.49 J cm^{-2} (n = 3 independent PUE samples). (c) Images of two self-healed PUE films pre- and post-peeling in lap-shear adhesion tests. Scale bar, 2 cm. (d) An adhesion strength-displacement curve of two self-healed PUE films showing adhesion strength of 18.43 ± 1.10 kPa (n = 3 independent PUE samples).

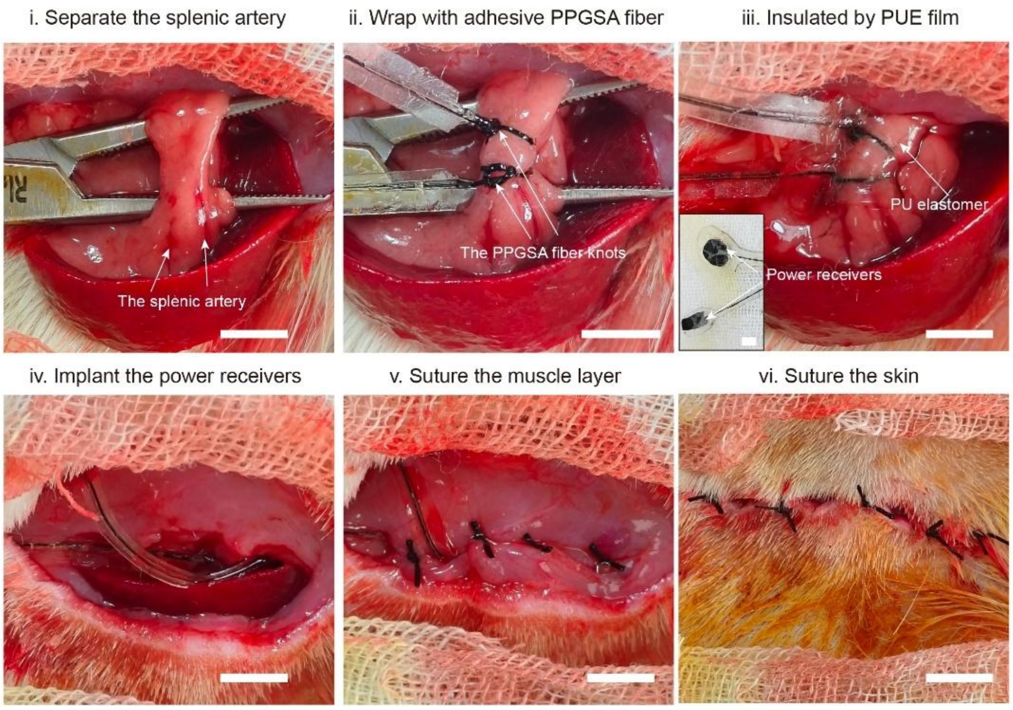

Figure S29. Surgical implantation procedure of SpNWS for splenic nerve stimulation. The surgical implantation of SpNWS comprises six sequential procedures: (i) anatomical isolation of the splenic neurovascular bundles (SNVBs), (ii) circumferential wrapping of PPGSA hydrogel fiber electrodes around SNVBs (the PPGSA hydrogel fiber was loosely knoted without inducing any stress compression on SNVBs), (iii) insulation encapsulation of PPGSA hydrogel fiber electrodes using a PUE film, (iv) subcutaneous implantation of wireless power-receiving modules, (v) layered muscular closure, and (vi) cutaneous suture. Scale bar, 5 mm.

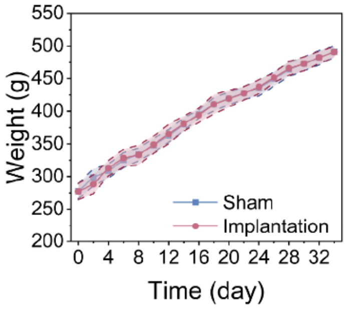

Figure S30. Body weight dynamics during 35-day SpNWS implantation. The SpNWS group showed no effect on body weight changes within 35 days post-implantation (n = 5 independent animals). Data are presented as the mean ± standard deviation.

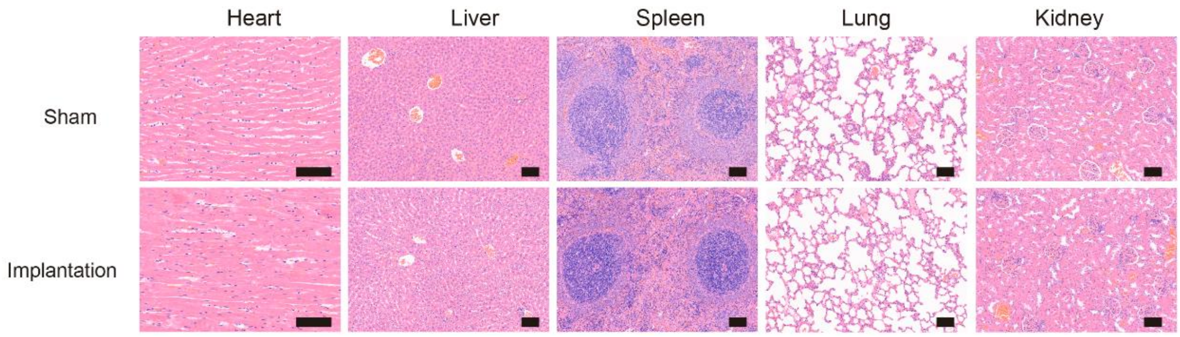

Figure S31. Histopathological assessment of major organ systems post-35-day SpNWS implantation. The SpNWS group showed no abnormalities in major organ H&E staining (heart, liver, spleen, lung, kidney) at 35 days post-implantation. Scale bar, 100 μm.

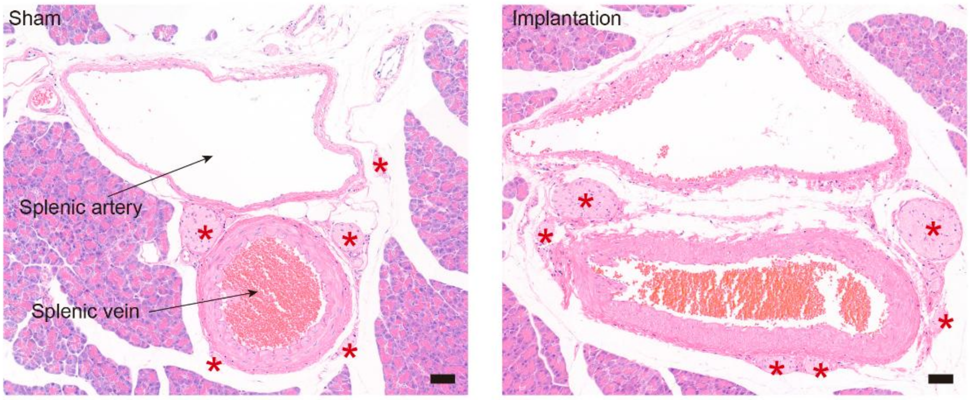

Figure S32. Histopathological assessment of the splenic nerve post-35-day SpNWS implantation. The SpNWS group exhibited no significant H&E abnormalities at the neurovascular bundle site 35 days post-implantation. The splenic artery and vein were anatomically labeled, while the splenic nerve was specifically demarcated with asterisks (*). Scale bar, 50 µm.

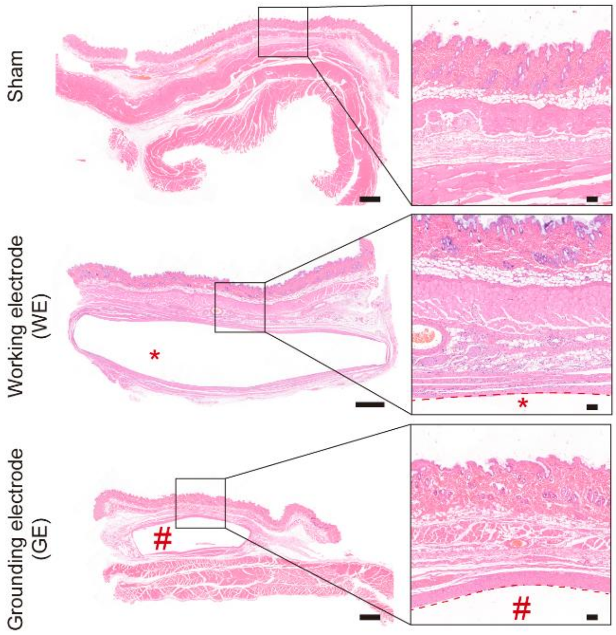

Figure S33. Histopathological assessment of power receiver-contacted skin post-35-day SpNWS implantation. H&E staining at working and ground electrode implantation sites revealed approximately 100 µm fibrous capsules at the electrode-tissue interface, with no other significant abnormalities. Working electrodes, ground electrodes, and electrode-skin boundaries were labeled with asterisks (*), hash symbols (#), and dashed lines, respectively. Scale bars, 500 µm (H&E images), 100 µm (H&E magnified images).

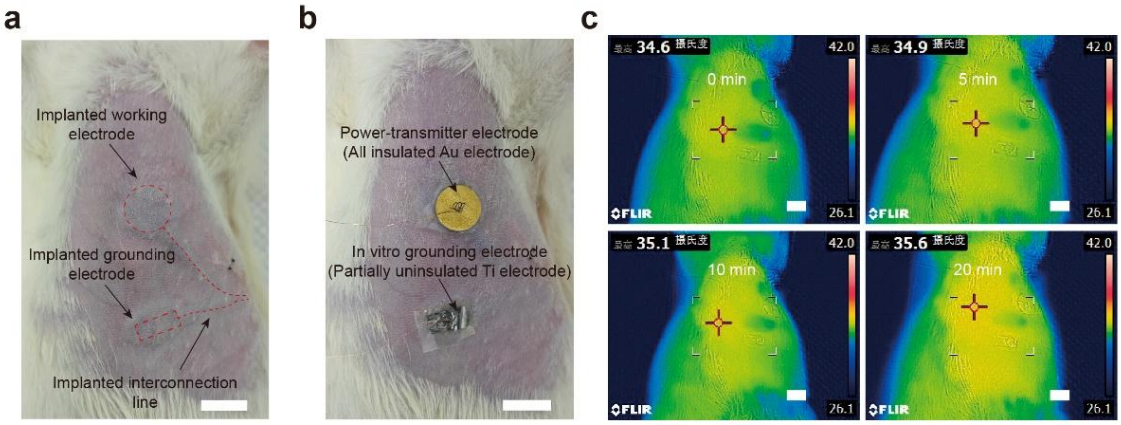

Figure S34. Thermal effect analysis of capacitive coupling. (a) Abdominal photograph of rat post-SpNWS implantation showing working electrode, grounding electrode, and interconnection line. Scale bar, 1 cm. (b) Abdominal photograph of rat post-SpNWS implantation with external electrodes for capacitive coupling. The external power-transmitter electrode should be optimally aligned with the implanted SpNWS neurostimulator's working electrode while ensuring full interfacial contact. The uninsulated area of the external grounding Ti electrode faces toward the uninsulated area of the implanted SpNWS neurostimulator's grounding electrode to achieve efficient electrical grounding. Scale bar, 1 cm. (c) Infrared thermographic images of rat abdomen during wireless capacitive-coupling stimulation, showing a temperature increase of 1 °C after 20-min stimulation. Scale bar, 1 cm.

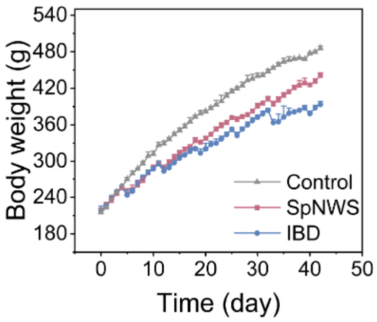

Figure S35. Body weight dynamics during SpNWS-based chronic splenic nerve stimulation. The SpNWS group showed less body weight reduction after each TNBS enema compared with the IBD group, demonstrating the anti-inflammatory efficacy of splenic nerve electrical stimulation (n = 8 independent animals). Data are presented as the mean ± standard deviation.

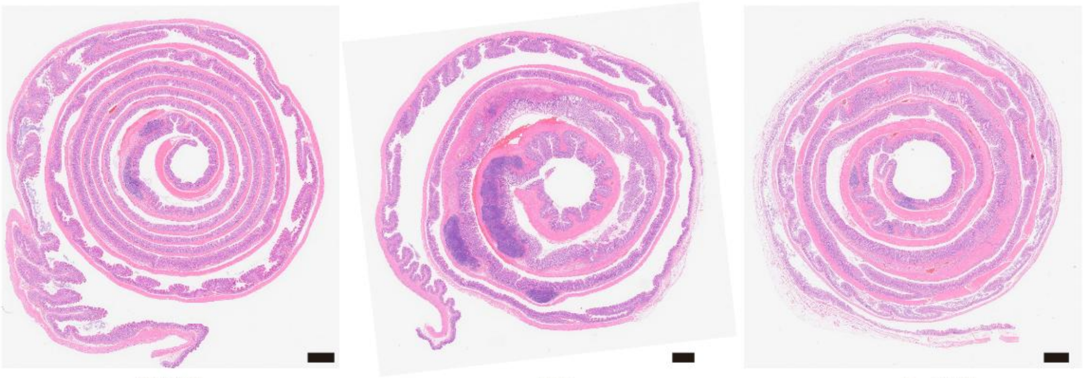

Figure S36. Histopathological assessment of colons after SpNWS chronic stimulation. The IBD group displayed transmural immune cell infiltration accompanied by goblet cell depletion in colonic tissues, whereas the SpNWS group presented only localized inflammatory cell clusters. Scale bar, 1 cm.

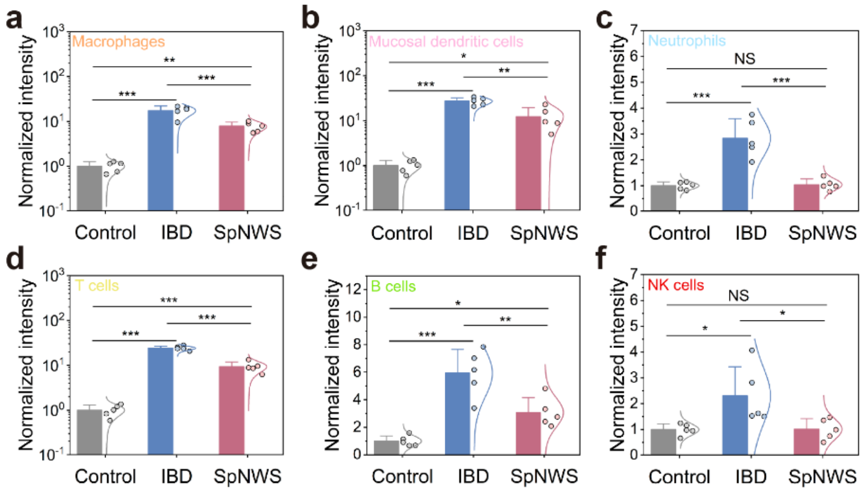

Figure S37. Immunofluorescence quantification of immune cells in colonic tissues from the control, IBD, and SpNWS groups. (a-f) Quantitative normalized fluorescence intensity comparison of CD68 (a), CD103 (b), MPO (c), CD3 (d),CD19 (e), and NKp46 (f) in colonic tissues from the control, IBD, and SpNWS in colonic tissues. The SpNWS group showed significantly lower normalized immunofluorescence intensities for CD68, CD103, MPO, CD3, CD19, and NKp46 in colonic tissues compared with the IBD group, indicating reduced infiltration of macrophages, mucosal dendritic cells, neutrophils, T cells, B cells, and NK cells (n = 5 independent animals). Data are presented as the mean ± standard deviation in (a-f) and were analyzed by one-way ANOVA first, and then by the Tukey's post hoc test. NS, not significant,$^*P \leq 0.05$, $^{**}P \leq 0.01$, $^{***}P \leq 0.001$. (a) $P_{\text{Control vs IBD}} = 4.06668 \times 10^{-6}$, $P_{\text{IBD vs SpNWS}} = 6.29169 \times 10^{-4}$. (b) $P_{\text{Control vs IBD}} = 9.094 \times 10^{-6}$, $P_{\text{IBD vs SpNWS}} = 0.00172$. (c) $P_{\text{Control vs IBD}} = 9.69437\text{E} \times 10^{-5}$, $P_{\text{IBD vs SpNWS}} = 1.19028 \times 10^{-4}$. (d) $P_{\text{Control vs IBD}} = 1.96232 \times 10^{-9}$, $P_{\text{IBD vs SpNWS}} = 2.28531 \times 10^{-7}$. (e) $P_{\text{Control vs IBD}} = 7.47975 \times 10^{-9}$, $P_{\text{IBD vs SpNWS}} = 0.00668$. (f) $P_{\text{Control vs IBD}} = 0.03077$, $P_{\text{IBD vs SpNWS}} = 0.03245$.

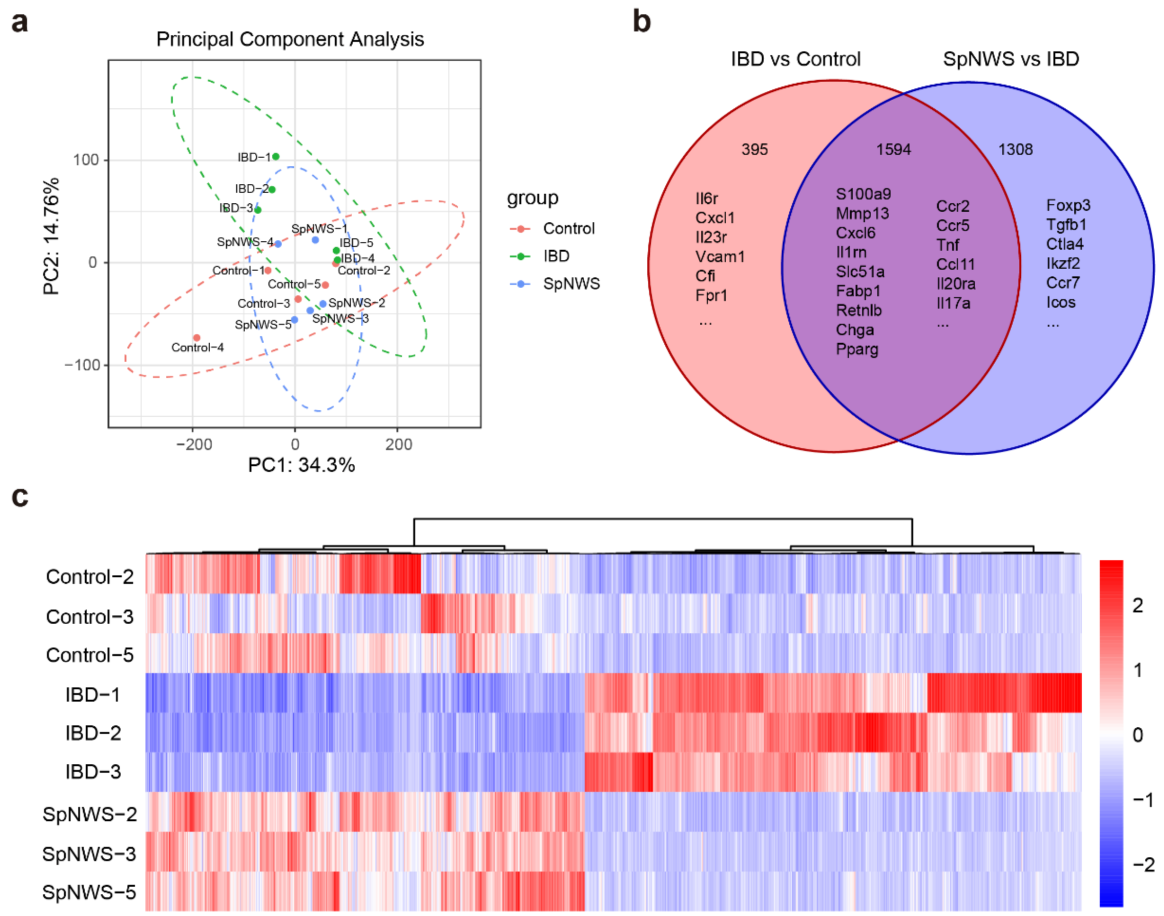

Figure S38. Bioinformatics analysis of RNA sequencing of rat colons from the control, IBD, and SpNWS groups. (a) Principal component analysis (PCA) diagram of the control, IBD, and SpNWS groups. (b) Venn diagram of differentially expressed genes (DEGs) between IBD versus control and SpNWS versus IBD. (c) Cluster heat map of DEGs common to both IBD versus control and SpNWS versus IBD comparisons.

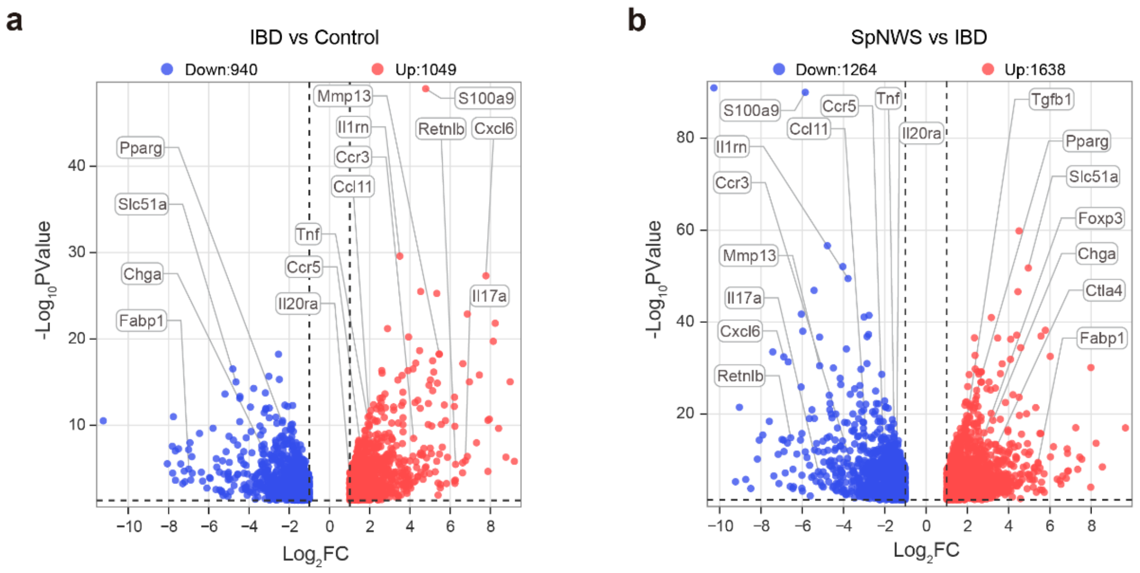

Figure S39. Analysis of the DEGs in IBD versus control and SpNWS versus IBD. (a, b) Volcano plots of the DEGs of IBD vs control (a) and SpNWS vs IBD (b). DEGs upregulated in IBD versus control included *S100a9*, *Il17a*, *Tnf*, and *Ccr3*, while downregulated DEGs included *Pparg*, *Chga*, and *Fabp1*. In SpNWS versus IBD, upregulated DEGs included *Foxp3*, *Ctla4*, and *Pparg*, whereas downregulated DEGs comprised *Il17a*, *Tnf*, *S100a9*, and *Ccr3*.

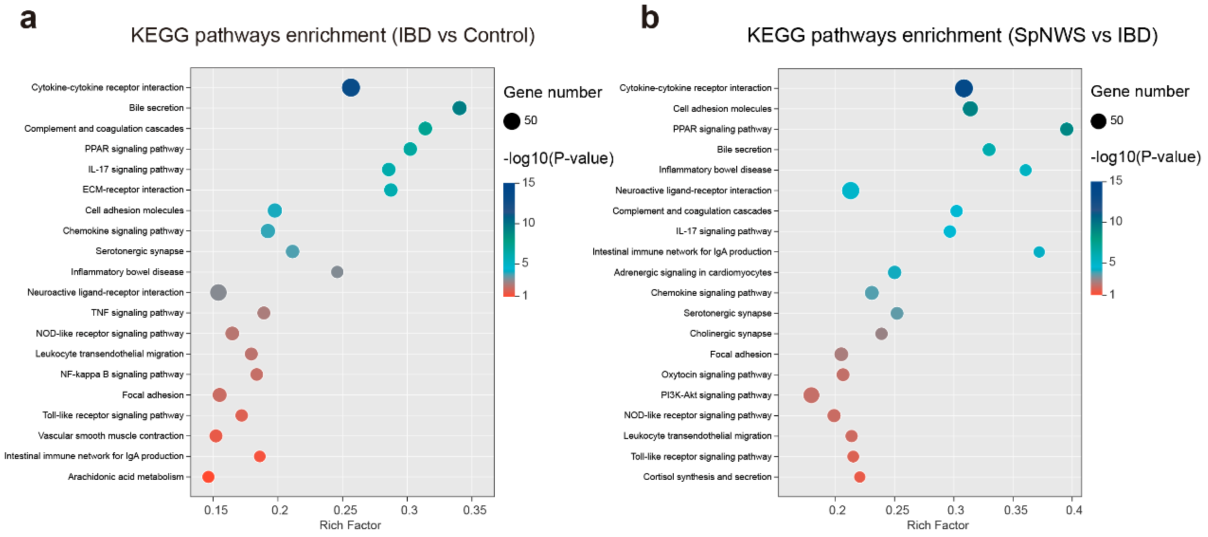

Figure S40. Differentially expressed genes enrichment analysis of the control, IBD, and SpNWS groups. (a) Kyoto encyclopedia of genes and genomes (KEEG) pathway enrichment bubble plot of the differentially expressed genes (IBD versus control). (b) KEEG pathway enrichment bubble plot of the differentially expressed genes (SpNWS versus IBD).

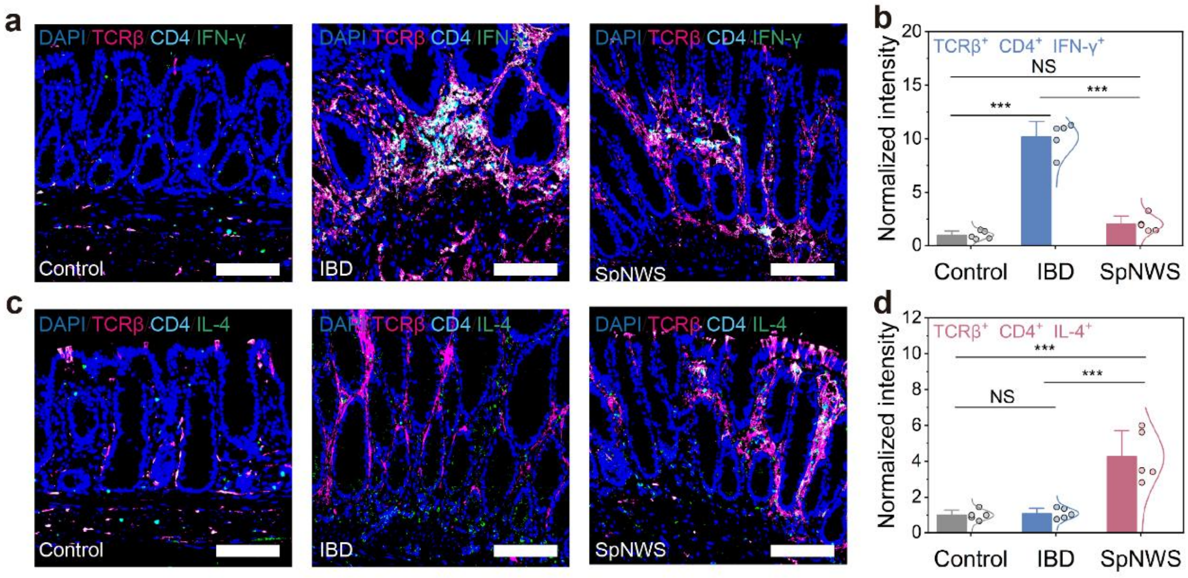

Figure S41. Immunofluorescence analysis of T_H1 and T_H2 cells in colonic tissues from the control, IBD, and SpNWS groups. (a) Representative immunofluorescence staining of T_H1 cells in colonic tissues from the control, IBD, and SpNWS groups. Scale bar, 100 μm. (b) Normalized fluorescence intensity of T_H1 ($TCR\beta^+$, $CD4^+$, IFN-γ^+) cells in colonic tissues from the control, IBD, and SpNWS groups. T_H1 cell populations were significantly reduced in SpNWS compared with IBD (n = 5 independent animals). (c) Representative immunofluorescence staining of T_H2 cells in colonic tissues from the control, IBD, and SpNWS groups. Scale bar, 100 μm. (d) Normalized fluorescence intensity of T_H2 ($TCR\beta^+$, $CD4^+$, IL-4^+) cells in colonic tissues from the control, IBD, and SpNWS groups. T_H2 cell populations were significantly increased in SpNWS compared with IBD (n = 5 independent animals). Data in (b) and (d) are presented as the mean ± standard deviation and were analyzed by one-way ANOVA first, and then by the Tukey's post hoc test. NS, not significant,*$P \leq 0.05$, **$P \leq 0.01$, ***$P \leq 0.001$. (b) $P_{\text{Control vs SpNWS}} = 0.27654$, $P_{\text{Control vs IBD}} = 1.66781 \times 10^{-8}$, $P_{\text{IBD vs SpNWS}} = 6.46231 \times 10^{-8}$. (d) $P_{\text{Control vs SpNWS}} = 1.73447 \times 10^{-4}$, $P_{\text{Control vs IBD}} = 0.98483$, $P_{\text{IBD vs SpNWS}} = 2.24072 \times 10^{-4}$.

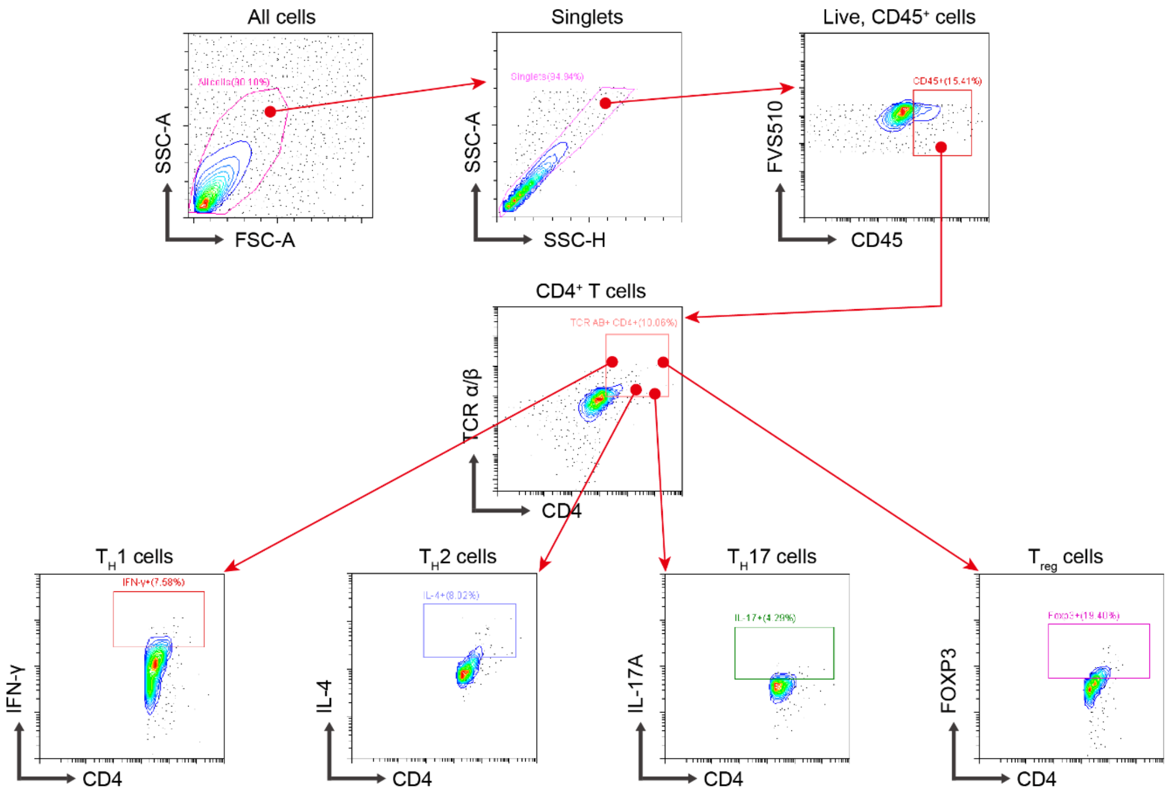

Figure S42. Flow cytometry analysis process of $CD4^+$ T cell subtypes. $CD45^+$ immune cells were initially isolated, followed by fluorescence-activated sorting of $CD4^+$ TCR α/β^+ T cells, ultimately yielding IFN-γ^+ (T_H1 cells), IL-4^+ (T_H2 cells), IL-17^+ (T_H17 cells), and $FOXP3^+$ (T_{reg} cells) T cells.

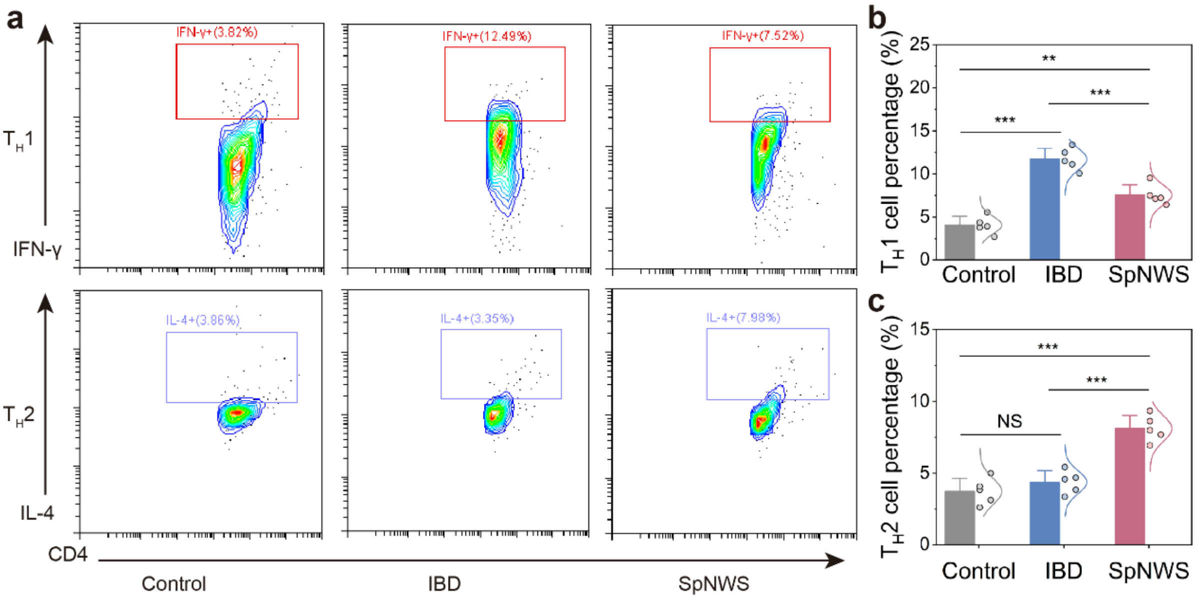

Figure S43. Flow cytometric analysis of colonic T_H1 and T_H2 lymphocyte subsets. (a-c) Fluorescence-activated cell sorting (FACS) plots (a) and quantitative comparisons of colonic T_H1 (b) and T_H2 (c) cell proportions across the control, IBD, and SpNWS groups. These results showed significantly reduced T_H1 proportions and increased T_H2 frequencies in SpNWS compared with IBD (n = 5 independent animals). Data are presented as the mean ± standard deviation in (b, c) and were analyzed by one-way ANOVA first, and then by the Tukey's post hoc test. NS, not significant,*$P \leq 0.05$, **$P \leq 0.01$, ***$P \leq 0.001$. (b) $P_{Control\ vs\ SpNWS} = 0.00123$, $P_{Control\ vs\ IBD} = 5.77852 \times 10^{-7}$, $P_{IBD\ vs\ SpNWS} = 2.8901 \times 10^{-4}$. (c) $P_{Control\ vs\ SpNWS} = 8.99641 \times 10^{-7}$, $P_{Control\ vs\ IBD} = 0.11537$, $P_{IBD\ vs\ SpNWS} = 1.2481 \times 10^{-6}$.
